# Substrate Exclusion Greenlights Physical Autocatalysis of Enzyme Activity in Membraneless Proto-Organelles

**DOI:** 10.1021/acs.biomac.5c00926

**Published:** 2025-10-29

**Authors:** Tasdiq Ahmed, Adya Verma, Shuichi Takayama

**Affiliations:** † Wallace H Coulter Department of Biomedical Engineering, 1372 Georgia Institute of Technology and Emory University, Atlanta, Georgia 30332, United States; ‡ Petit Institute for Bioengineering and Bioscience, Georgia Institute of Technology, Atlanta, Georgia 30332, United States

## Abstract

Cells modulate phase separation to control condensate formation, yet how such organelles affect enzyme activity is poorly understood. This paper describes how substrate-excluding, membraneless proto-organelles can increase enzyme mobility while also controlling the extent of reaction acceleration. The model system—an equimolar polyelectrolyte-nucleotide coacervate—allows for probing compositional influence on the activity of the biopolymer-processing enzyme dextranase. Increasing the phase-forming constituent concentrations is sufficient to sustain fast intradroplet dextranase mobility and enhance hydrolysis, even when placed in highly viscous, concentrated regimes of the organelle-excluded substrate dextran. This catalytic uptick is mediated largely by organelle material properties, eschewing the need for effective substrate or enzyme concentration enrichment. Physical analysis reveals that dextranase dimensions fall within the organelle mesh size range and that constituent concentrations induce changes in droplet viscosity. Enzyme diffusion measurements within polymer solutions that mimic the intracellular/prebiotic landscape underscore the catalytic, and potentially evolutionary, advantage of compartments, enabling faster enzyme mobility.

## Graphical abstract

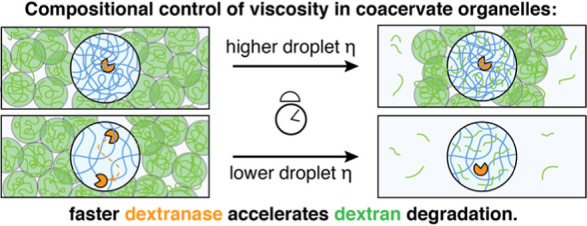

## 1 Introduction

The spontaneous self-assembly of oppositely charged polyelectrolytes into membraneless coacervate droplets has long been theorized as a mechanism by which early cells came into existence.^1^ These droplets likely served as spatially distinct zones for biochemical reactions to occur, such as the production or degradation of nucleic acids and polysaccharides, molecules that can retain and transmit information or have supporting roles in structure and energy. Coacervates form by liquid–liquid phase separation (LLPS), a process also important for the formation of biomolecular condensates, suggesting a link between the development of functionalized liquid-like structures in early life and the crucial membraneless organelles found today.

Compartmentalization of biopolymers into various intracellular phase-separated bodies has been increasingly recognized within the past two decades to be crucial for cellular organization and function.^2,3^ Rheological characterization of biomolecular condensates has strongly hinted at the critical role that control over droplet composition may have in regulating cell function.^4,5^ Uptake of enzymes into condensates, often formed from proteins and/or nucleic acids undergoing coacervation, has been linked to increases in catalytic activity.^6^ The general belief is that partitioning of substrate into condensates increases their local concentration, leading to a higher probability of catalysis as enzyme and substrate become colocalized.^7^ This is supported by evidence that forming smaller compartments forces enzyme and substrate to interact as space is condensed, leading to a lower apparent substrate *K*_m_ and greater activity.^8^ However, this is not entirely reflective of nature. Substrates could be excluded from specific compartments such as within the nucleolus; additionally, nucleoli in cancer cells are often enlarged^9^ and the size directly correlates with frequency of nucleolar activities such as rRNA transcription.^10^ This article presents an alternative view to the above concepts by considering other possibilities in the development of functionalized centers in early life, termed proto-organelles. For such a stance, we view proto-organelles and modern-day condensates through a material lens rather than a concentration- or size-specific lens. We use an LLPS-formed proto-organelle where material properties such as internal viscosity and mesh size, rather than enzyme concentration, result in increased enzyme activity; we demonstrate how the model system is not reliant on increases of effective substrate concentration as the substrate is instead excluded from the enzyme-enriched proto-organelle.

It is thought that modulating the diffusion of enzyme and/or substrate is a process cells exploit, but even in in vitro models, this has not been fully tested.^6,11^ Work until now has considered the effects of varying substrate^12^ concentrations or using different protein droplets with differing viscosities^13^ but they do not address the question of whether the change in the composition of a single type of condensate leads to changes in enzyme activity, much less diffusion. Because intracellular droplets can be regulated by reversible dissolution and condensation,^3^ there may be changes largely in constituent concentrations over a set period of time, rather than incorporating or removing different types of polymers. As such, demonstrating whether control over droplet composition may regulate enzyme diffusion and function should have significant physiological implications. Our system as previously studied^14^—dextranase as the model enzyme, the polymer dextran 500 K as the model substrate, and coacervates formed by adenosine triphosphate (ATP) and the polycation poly­(diallyldimethylammonium chloride) (PDDA) as the model proto-organelle—is suitable for testing the questions of droplet composition and the interplay with enzyme mobility and catalysis. Similar systems have been widely reported in the literature.^15–21^ We form coacervates at the commonly used fixed ratio of 1:1 ATP/PDDA, where concentrations are monomer equivalent. Importantly, by only changing the concentration within a set range, as feasible in prebiotic environments via wet–dry cycling,^22–24^ we significantly alter coacervate physical properties such that we can relate proto-organelle composition to enzyme mobility and function. Furthermore, the dextran 500 K substrate is significantly barred from entering the proto-organelles. Dextran has been recently shown to adsorb to a coacervate interface, forming a semipermeable membrane for enzyme-loaded protocells that can allow for complex assemblies;^25,26^ dextranase was used to modulate the stability of the membranized droplets. The coacervate system used in this work likely exhibits different surface properties, preventing such membranization and dextran entry. Our substrate-excluded model is in contrast to recent publications that study droplets where enzyme and substrate are colocalized,^13,27,28^ a condition not reflective of some condensates like transcriptional granules.^29^ Using a model proto-organelle system, we analyze enzyme mobility within a range of proto-organelle compositions. These findings allow us to assess proto-organellar enzyme activity, specifically for the ability to degrade an organelle-excluded substrate. We complement the work by elucidating the material properties of the coacervate base of our proto-organelle, give experimental consideration to the substrate’s polymeric nature, and finally outline a theoretical treatment of the alternative strategies afforded by substrate exclusion. This work significantly expands upon the previous finding that compartmentalization alleviates substrate inhibition by diving into the molecular picture to mechanistically understand how uptake of an enzyme into a droplet is beneficial and how it may be leveraged in nature.

## 2 Materials and Methods

### 2.1 Materials

Dextranase, the polymers poly­(ethylene glycol) (*M*_w_ 3350; 35,000; 100,000; 400,000) and dextran (*M*_w_ 10,000; 70,000; 500,000), ATP, MES, and fluorescent probes FITC-dextran (*M*_w_ 10,000; 20,000; 40,000; 500,000) and FITC-Ficoll (*M*_w_ 400,000) were purchased from Sigma. Atto488-COOH and Atto655-COOH were purchased from ATTO-TEC. Poly­(diallyldimethylammonium chloride) (PDDA, *M*_w_ 240,000) was purchased from Polysciences. Solutions of PDDA were prepared with respect to the monomer concentration. Buffers for all experiments were prepared as 20 mM MES in water at pH 6.0 and were filtered using 0.22 μm pore-size membranes (EMD Millipore). Dextranase was labeled with Atto647N-NHS ester or Atto655-NHS ester (ATTO-TEC) following manufacturer protocol. The labeled enzyme was then purified using Slide-A-Lyzer G3 dialysis cassettes (Thermo Fisher) over 3 days, with the exchange medium replaced with Millipore-filtered water each day. Degree of labeling was confirmed using a NanoDrop spectrophotometer to be approximately 2 dye molecules per enzyme.

### 2.2 Fluorescence correlation spectroscopy

Dye-labeled enzyme was prepared for all FCS experiments, unless specified otherwise, at a final concentration of approximately 2 μg mL^–1^. Samples were pipetted into individual wells of an 18-well glass bottom slide (ibidi, #81817), with 0.05% Tween20 added in noncoacervate cases to prevent protein aggregation and sticking to the coverslip. Experiments were conducted at room temperature, set as (23.0 ± 0.5) °C. Intensity traces were collected for 15–60 s using an Abberior Facility Line STED instrument with a pulsed 640 nm laser and TimeHarp 200 TCSPC electronics (PicoQuant). The laser power was optimized at the time of collection to maximize the intensity but limit saturation of the observation volume. Time traces were then imported into SymPhoTime (PicoQuant) to generate autocorrelation traces via the Grouped FCS function for each replicate set (at least *N* = 10–16) and were also fitted in software. Atto655 solutions in nanomolar amounts (and with 0.05% Tween20) were used to calibrate the observation volume necessary for diffusion coefficient calculations, using temperature-corrected dye diffusivities, as reported by PicoQuant. Imaging 20 nm Crimson beads in x–z gave reasonable confirmation of the fitted values. During collection, the focal plane was set to within a few micrometers above the coverslip. Fitting was performed with a 3D autocorrelation fitting function that incorporated one or two diffusive component(s) and one triplet state in which the calibration-derived time was found to be ∼25 ns. For each group of replicate traces, the same fit function was applied to all curves simultaneously and not adjusted further. In some cases, although uncommon, the curve was excluded if photobleaching could be observed in the intensity trace, showing an irregularly shaped autocorrelation trace. The anomaly parameter was found to be largely noninfluential in fitting and was thus fixed to 1 to reduce the number of free fitting parameters. Fitted data were exported and compiled in GraphPad Prism.

### 2.3 Partition Coefficient Determination

In preparation of partition experiments, untreated 8-well high glass bottom slides (ibidi, #80801) were plasma treated for 4 min before placing in a chamber containing a few drops of (tridecafluoro-1,1,2,2-tetrahydrooctyl) trichlorosilane (Gelest, product SIT8174.0) that would vaporize upon vacuum and subsequently covalently bond to the glass. After 2 h of deposition, slides were removed from the chamber and immersed immediately into a Coplin jar containing 1% Pluronic-F127 in water. Slides were kept in the jars for at least 24 h and would be washed profusely with purified water before use. This treatment would prevent droplet wetting and dextran from adsorbing onto the glass surface. For the partition experiment, we injected at the final concentration 50 μg mL^–1^ of FITC-dextrans (10k, 20k, 40k) and free Atto488 dye into freshly prepared suspensions of 50 mM and 100 mM coacervates. Droplets were allowed to settle on the well surface for at least 1–2 h before imaging. Images were collected by using a Nikon W1 spinning disk confocal microscope. Fluorescent intensities were measured using ImageJ (NIH) and were corrected for background both in and out of the droplets.

### 2.4 Degradation Assay

To test for dextranase dissolution of a dextran drop embedded in a well of PEG, we followed our group’s protocol as previously published, with some modifications.^14^ Briefly, 6 μL of 20 wt % dextran 500 K spiked with ∼0.2 wt % FITC-dextran 500 K (TdB Laboratories) in MES pH 6 was pipetted into the center of wells in a 96-well, clear flat bottom/black wall, surface-nontreated plate (Corning, #3631). Next, 1 μL of the ATP/PDDA coacervate loaded with unlabeled dextranase at a concentration of 100 μg mL^–1^ were injected directly into the dextran droplet. Coacervate concentrations were at 25, 50, and 100 mM ATP/PDDA. Dextran droplets injected with enzyme-free coacervates or mass-corrected amounts of enzyme in buffer were used as controls. Finally, 200 μL of a 10 wt % PEG 35K solution in MES pH 6 was gently pipetted atop the dextran droplets. The plate was then transferred to an EVOS FL automated microscope (ThermoFisher) set to record time-lapse fluorescence (GFP channel) images at 2× magnification, in 1 h intervals over the course of 24 h. Stitched images of the wells were exported upon completion. The image sets were then analyzed for area using the *ComponentMeasurements* function in Mathematica (Wolfram Research); no manual image processing was performed for the analysis. In the 100 mM ATP/PDDA condition, at time points where the droplet was near total dissolution, a broad threshold failed to identify the dextran droplet; in these cases, droplets were manually measured for area using ImageJ. Because of low-surface tension between stably demixed PEG and dextran,^30,31^ the dextran droplets would flatten; areas for each droplet were then normalized against the highest measured area rather than initial area.

### 2.5 Cantilever Viscometry

Viscosity of the ATP/PDDA coacervates was measured using an AFM-based formalism developed previously.^32,33^ Briefly, as micron-sized cantilevers experience thermal fluctuations characterized by a resonant frequency, monitoring resonance dampening by a viscous fluid can in turn be used to calculate the fluid’s viscosity. Coacervates with concentrations of 60, 80, and 100 mM ATP/PDDA were freshly prepared, spun down, isolated, and transferred to a facility housing a Dimension Icon AFM (Bruker). After loading a probe with reflective aluminum coating (MikroMasch, HQ:NSC15/Al BS, nominal force constant 40 N m^–1^) onto a fluid probe holder, approximately 20 μL of a coacervate was carefully micropipetted onto the probe stage, engulfing the probe. After the optics were calibrated, resonant frequencies were measured in the accompanying Nanoscope software using the high-speed data capture feature. Data were also collected for the probe in air and in water for later analysis. The room temperature at time of experiment was 15.67 °C. Amplitude vs frequency data were binned at 763 Hz. After performing a Gaussian fit to the average of 11 measurements, resonant frequencies could be extracted. Viscosities were calculated using an equation from Chen et al.^32^

$$\omega =\frac{1}{8}\left(\sqrt{9{\left(K\eta \rho \right)}^{4}+64\omega_{0}^{2}}-3K^{2}\right)$$

where ω is the fluid resonant frequency, ω_0_ is the resonant frequency in air, *K* is a constant specific to the AFM probe, η is the fluid viscosity, and ρ is the fluid density. *K* was first calculated using the measured frequencies for water (175.0 kHz) and air (259.1 kHz) and known as η and ρ for water at the indicated temperature. Coacervate density could not be easily measured given small sample volumes, so assuming a density of 1.1 g mL^–1^, viscosities could be estimated using the above equation. Viscosities were separately calculated using the Stokes–Einstein equation, inputting the dextranase diffusion means found through FCS. Given viscosity is temperature-dependent, and the FCS measurements were performed about 7–8 °C higher than the AFM measurements, a simple correction was performed to allow comparison of the two methods, using the viscosity of water at 23 and 15.67 °C. By multiplying a ratio of the two values to the AFM-derived coacervate viscosities, a rudimentary correction could be applied.

## 3 Results and Discussion

### 3.1 Dextranase Diffusion within a Model Proto-Organelle

Dextranase is a hydrolytic enzyme typically found in bacteria, and it most commonly cleaves the (1 → 6)-α-glycosidic linkages of its polysaccharide substrate dextran (Figure 1a). It is known that dextranases tend to exhibit higher affinity (lower *K*_m_) with increasing dextran molecular weight.^34–36^ Enzyme performance is gauged by calculating the ratio of turnover rate to the Michaelis constant, *k*_cat_/*K*_m_, where a very high *k*_cat_/*K*_m_ (≥10^8^ M^–1^ s^–1^) indicates that the enzyme is diffusion limited.^37^ In this state, the enzyme is said to be “catalytically perfect” and is limited in its activity only by the diffusivity of the enzyme and/or substrate. Using published data^38^ on an isolated endodextranase, one can calculate *k*_cat_/*K*_m_ to be 10^8^ M^–1^ s^–1^ for dextran 2000 kDa (Figure S1a). The values suggest that dextranase is a diffusion-limited enzyme for large (≥500 kDa) molecular-weight dextrans. For such enzymes, low diffusivity of the substrate or the enzyme decreases turnover. In crowded environments, such as the intracellular space or solutions of bulky polymers, this may pose a challenge for efficient catalysis (Figure S1b).

**Figure 1:**
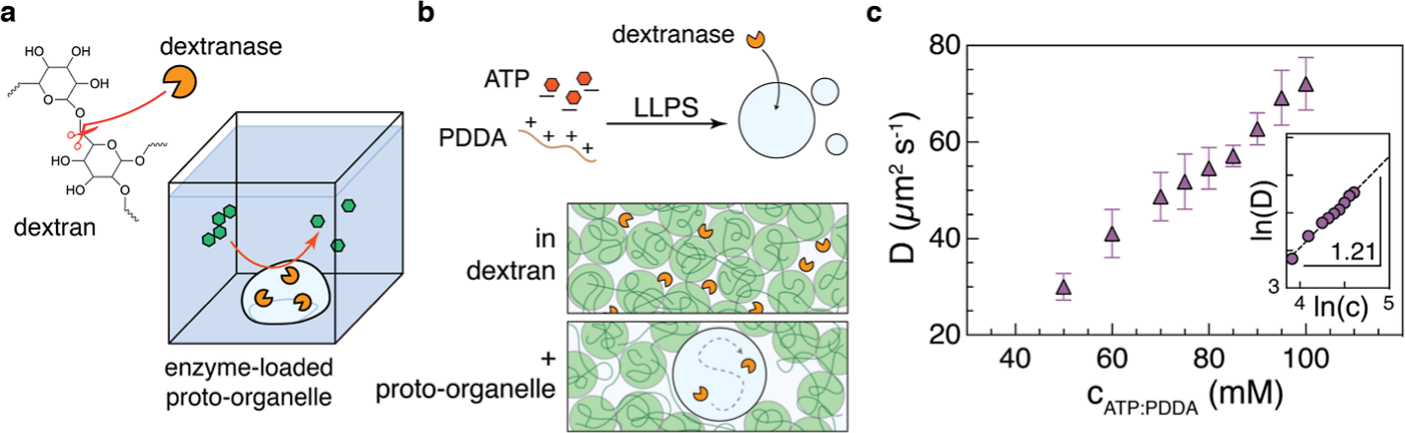
Proto-organelle composition dictates dextranase mobility. (a) (left) Dextranase, an enzyme typically sourced from bacteria, specifically hydrolyzes glycosidic linkages in the natural, branched polysaccharide dextran. (right) Schematic of a dextranase-loaded proto-organelle facilitating catabolism of condensate-excluded polysaccharide. (b) (above) Negatively charged ATP and the polycation PDDA undergo liquid–liquid phase separation (LLPS), forming coacervate droplets that are the basis of a proto-organelle. Dextranase when in solution migrates into the coacervates, establishing a proto-organelle. (below) In a semidilute or concentrated solution of dextran, dextranase may be slowed down by both space limitations and substrate binding. By housing enzyme within a proto-organelle that excludes dextran, dextranase could be lifted from such restrictions. (c) Diffusion data of Dextranase-Atto655 when in a coacervate of specified composition. The inset shows data means converted to the log–log format and fitted to a linear regression giving a scaling exponent of 1.21 such that *D* ∼ c^6/5^. Data shown as mean ± CI, *n* = 14–16 measurements.

Before testing ways of increasing dextranase diffusion, we estimated its free diffusion coefficient to serve as a baseline. Measuring diffusion of the enzyme within a crowded environment would be difficult without labeling, and so we sought to fluorescently label a commercially sourced dextranase and perform FCS experiments on the labeled enzyme in order to extract diffusivities in free buffer, polymeric solutions, and the coacervates. While protein labeling is often a crucial step in many studies, dye selection becomes paramount even beyond consideration of its performance in microscopy.^39^ Although Atto dyes are desired for their exceptional photostability, Atto647N, considered a benchmark dye for single-molecule applications like FCS, is prone to aggregation and can associate strongly with hydrophobic substances such as lipids.^40^ We found that when attempting to partition dextranase labeled with Atto647N into the ATP/PDDA coacervates, the enzyme strongly localized to the droplet interface (Figure S2b). We found from a panel of dyes that Atto655 is predicted to be hydrophilic at pH 6 and thus was used for labeling. Indeed, replacing Atto647N with 655 was sufficient in redistributing enzyme through the droplet (Figure S2a). Performing FCS on Dextranase-Atto655 serially diluted to 5, 10, and 20 μg mL^–1^ allowed us to fit a linear regression that revealed a diffusion coefficient at infinite dilution of *D*_0_ = 79.7 μm^2^ s^–1^ (Figure S3). Using Stokes–Einstein, we determined the approximate dextranase hydrodynamic radius to be 2.92 nm at a temperature of 23 °C. These values would be used for reference in this study.

Knowing these key parameters for the dye-labeled dextranase, we sought to build a model with which we could study our questions regarding enzyme diffusion within a proto-organelle. Figure 1a shows the configuration of our model, in which a proto-organelle houses dextranase and is surrounded by a solution containing dextran, which is physically excluded from the droplet. We use as proto-organelles droplets formed by the coacervation of ATP and PDDA. Notably, these droplets are formed at a starting fixed ratio of 1:1 nucleotide/polymer, where we envision simply increasing the concentration, as possible via water evaporation in early life, impacts the organelle properties (Figure 1b). We confirmed that the enzyme both partitioned into the proto-organelle and stayed within the droplet even when surrounded by the substrate. Indeed, we observed for 3 h after injecting a coacervate loaded with Dextranase-Atto655 into a solution of dextran 500 K and saw no detectable leaking of enzyme out of the droplet (Figure S4). This is in line with our previous finding of strong partitioning of dextranase within these coacervates.^14^

Prior work had demonstrated that compartmentalization of dextranase into an ATP/PDDA coacervate improved the enzyme’s catalysis of dextran.^14^ Those observations were rationalized as physical exclusion of the substrate from an enzyme-rich domain could work to counter the biochemical phenomenon of substrate inhibition, where very high concentrations of the substrate inexplicably stymie enzyme activity. Delving into the molecular picture was envisioned to provide further insight, such as uncovering the influence enzyme mobility and polymer size and state may have on catalysis. We observed that the equimolar ATP/PDDA coacervates exhibit changing volume ratios, wherein rising initial concentrations resulted in larger coacervate phase volumes (Figure S5). Additionally, the larger volume coacervates could be pipetted easily, hinting at a change in viscosity (to be discussed in Section 3.3). This all suggested that material properties of the coacervate could be altered significantly just by changing constituent concentrations within an order of magnitude range, which are likely in prebiotic environments. It was then possible to examine how enzyme diffusivity changes with respect to the different formulations. We first anticipated that enzyme diffusivity should overall decrease with respect to increasing the initial PDDA concentration. However, we repeatedly saw that the enzyme diffusivity increased as the ATP/PDDA concentration increased; the relation from scaling was *D*_enz_ ∼ *c* ^6/5^ (Figure 1c). While we did not measure enzyme diffusion outside the range of 40–100 mM ATP/PDDA, we anticipate that above 100 mM, *D*_enz_ stabilizes at *D*_0_, and that below 40 mM, *D*_enz_ continues to decrease in accordance with coacervate bulk viscosity, which may or may not conform with our reported scaling relation. Both the change in volume and enzyme diffusion are consistent with effects of increasing charge imbalance due to increasing excess of negative ATP compared to the positive PDDA monomer.

### 3.2 Enzyme Activity Is Tuned by the Proto-Organelle Constituent Concentration

Because we uncovered a system linking enzyme mobility and coacervate concentration, we sought to exploit this link to induce the faster degradation of dextran. Our group previously tested one coacervate formulation, 25 mM ATP/PDDA, in its ability to unleash dextranase (which is confined to the coacervate) from substrate inhibition by excluded dextran but did not examine diffusion-optimized coacervate environments.^14^ To remedy this limitation, we repeated the dextran degradation experiment but injected a range of coacervates in which there were significant differences in the measured enzyme diffusivity (Figure 2a). Droplets at 100 mM ATP/PDDA still exhibited dewetting behavior (Figure S6) with similar contact angles as in the 25 mM formulation,^14^ so we could assume that droplets retained their shape during the experiment. In the experimental configuration, featuring a PEG-dextran aqueous two-phase system, we expect dextran to wet the coacervate surface like other polymers,^41–43^ facilitating reactions at the interface. Still, free chains may fluctuate into the droplet and engage in interior reactions. Degradation products of low-molecular-weight (<10 kDa) dextran should enter the coacervate, accumulating near the end of the reaction. Regardless, our interest is in the capabilities of faster-diffusing dextranase, and so we doubled the dextran concentration to 20 wt %, which our group previously observed to be a concentration that nearly eliminates the benefits compartmentalization has on dextranase activity. This benefit loss was reasoned to result from high viscosities of the substrate solution and coacervate interior, where low mobility of substrate and enzyme makes diffusion the rate-limiting factor. As we now expect enzyme diffusion to have been slow in the 25 mM ATP/PDDA formulation, then perhaps formulations allowing for faster enzyme movement may serve to counter the physical limitations of the substrate. We should note that 20 wt % dextran is expected to be just past the second crossover limit c** for dextran 500 K into the concentrated regime.^44^ Given water is a theta solvent for dextran, we would expect ξ ∝ *b*ϕ^–1^ where ξ is the mesh size (to be discussed in later sections), *b* is the Kuhn length, and ϕ is the volume fraction;^45^ as c* = 4 wt %, then there could be a significant drop in ξ such that at c**, enzyme movement is severely limited, likely to be 100-fold slower than *D*_0_ based on Stokes–Einstein predictions. Still, the proto-organelle housing the enzyme should release the enzyme from this dextran-imposed restriction, so we deduce that tailoring the coacervate mesh size to suit the enzyme should lead to more efficient degradation. Note that the dextran 500 K substrate is about 5–7 times larger radius wise than the enzyme, so modulating mesh size for the enzyme should not lead to substrate entering the proto-organelle.

**Figure 2:**
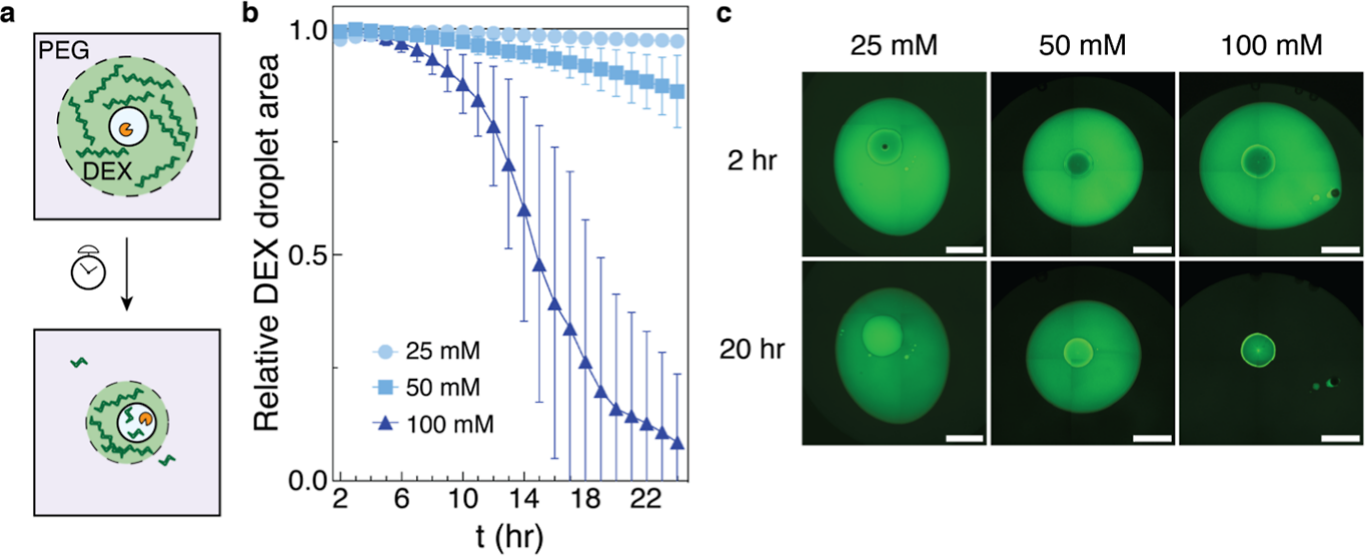
Altering proto-organelle composition to increase enzyme diffusion leads to faster substrate degradation. (a) PEG and dextran (DEX) form an aqueous two-phase system which preserves droplet concentration and prevents dehydration. Upon injection of a proto-organelle, dextran begins to hydrolyze and the droplet shrinks. Degradation end products (small fluorescent isomaltose oligomers) accumulate in the coacervate over time. Tabulating droplet area over time allows for a quantitative analysis of dextranase activity. (b) Dextran droplets shrink faster when injected with proto-organelle of higher [ATP/PDDA]. Enzyme was added to the isolated coacervate phase (final concentration 100 μg mL^–1^) before 1 μL of the enzyme-loaded proto-organelle was injected into 6 μL of 20 wt % dextran 500 K spiked with FITC-dextran 500 K and subsequently overlaid with 200 μL of 10 wt % PEG 35 K, all in 20 mM MES pH 6. Wells were monitored for 24 h after injection. Proto-organelles were set at the equimolar [ATP/PDDA] conditions of 25 mM, 50 mM, and 100 mM. Data shown as mean ± SD, *n* = 20 per condition; lines connect mean values for each time point. (c) Representative stitched fluorescent images showing dextran droplet shrinking upon injection of a proto-organelle. Image brightness and contrast were processed individually in this figure for easier visualization. Scale bars, 1 mm.

Excitingly, we found that the dextran droplet into which the enzyme-laden proto-organelle was injected would disintegrate faster in accordance with increasing enzyme diffusivity (Figure 2b,c). Area shrinking was noticeably faster over time, possibly confirming the effects of substrate viscosity, where lower concentrations of dextran—as facilitated by dextranase—lead to decreasing dextran viscosity. The 100 mM ATP/PDDA proto-organelle led to the fastest degradation, in which 70% of the replicates reached complete dextran dissolution well within 24 h. Returning to the previous 25 mM ATP/PDDA, we saw little droplet shrinking, as expected based on previous findings that compartmentalized (or free^46^) dextranase has negligible activity enhancement when in 20 wt % dextran 500 K. At 50 mM ATP/PDDA, there is an improvement in catalysis, but it is still far off from the highest concentration coacervate as dextranase diffusivity in the 50 mM formulation is around half of that in 100 mM ATP/PDDA.

The clear increase in the degradation rate corresponding to an increase in diffusion is in line with our reasoning that providing a route for a diffusion-limited enzyme to overcome the locally viscous environment can have profound effects on its activity. This then expands our previous finding that compartmentalization alleviates substrate inhibition to further include that compartmentalization within proto-organelles and condensates can provide an enhanced microenvironment that aids an enzyme in sustaining its maximal diffusion and activity (and, in theory, approach catalytic perfection) despite interference from viscous substrate solutions.

### 3.3 Mechanistic Underpinning of Diffusion Effects

We then shifted focus toward uncovering a physical basis to explain the results above. One useful physical parameter, the characteristic mesh size ξ of a polymer solution, can be estimated by using nanometer-sized fluorescent probes. Initial testing with FCS on fluorescent dextrans of varying hydrodynamic radii at dilute concentrations (∼1 to 10 μg mL^–1^) revealed that sub-4 nm radii probes experience an apparent viscosity slightly above that of a small dye (Figure S7). This suggested that ξ could be in the range of dextranase’s radius, but a strong approximation of how ξ scales to coacervate formulation (or possibly factors like virial coefficients) would be difficult to achieve with the probes available. We then performed simple partition experiments to readily estimate ξ and where dextranase fits in (Figure 3a). Using the same FITC-dextrans, we imaged 50 and 100 mM ATP/PDDA droplets about 1–2 h after incubation with the probes. From these images, we measured intensities and computed partition coefficients *K* where *K* > 1 represented greater partitioning into the dense coacervate phase. Measuring the zeta potential of the probes showed that they were not significantly charged (Figure S8), so charge could be ruled out as a major confounding factor. The results showed that dextran 10 K (*R* ≈ 2.3 nm) would enter the droplets, but dextran 20 K (∼3.3 nm) would generally be excluded (Figure 3b,c). Dextranase’s radius (2.9 nm) falls within the range bookended by these two probes. Given that the enzyme partitions into the droplets at these concentrations, we could suggest that the true ξ for ATP/PDDA coacervates is slightly above 2.9 nm. Also of note is the increase in fluorescence observed within the proto-organelles at later time points in the degradation experiments (Figure 2c), partially corroborating the mesh size results, in that lower-molecular-weight dextran produced in catalysis may enter the proto-organelle, whereas the original large dextran 500 K substrate is excluded.

**Figure 3:**
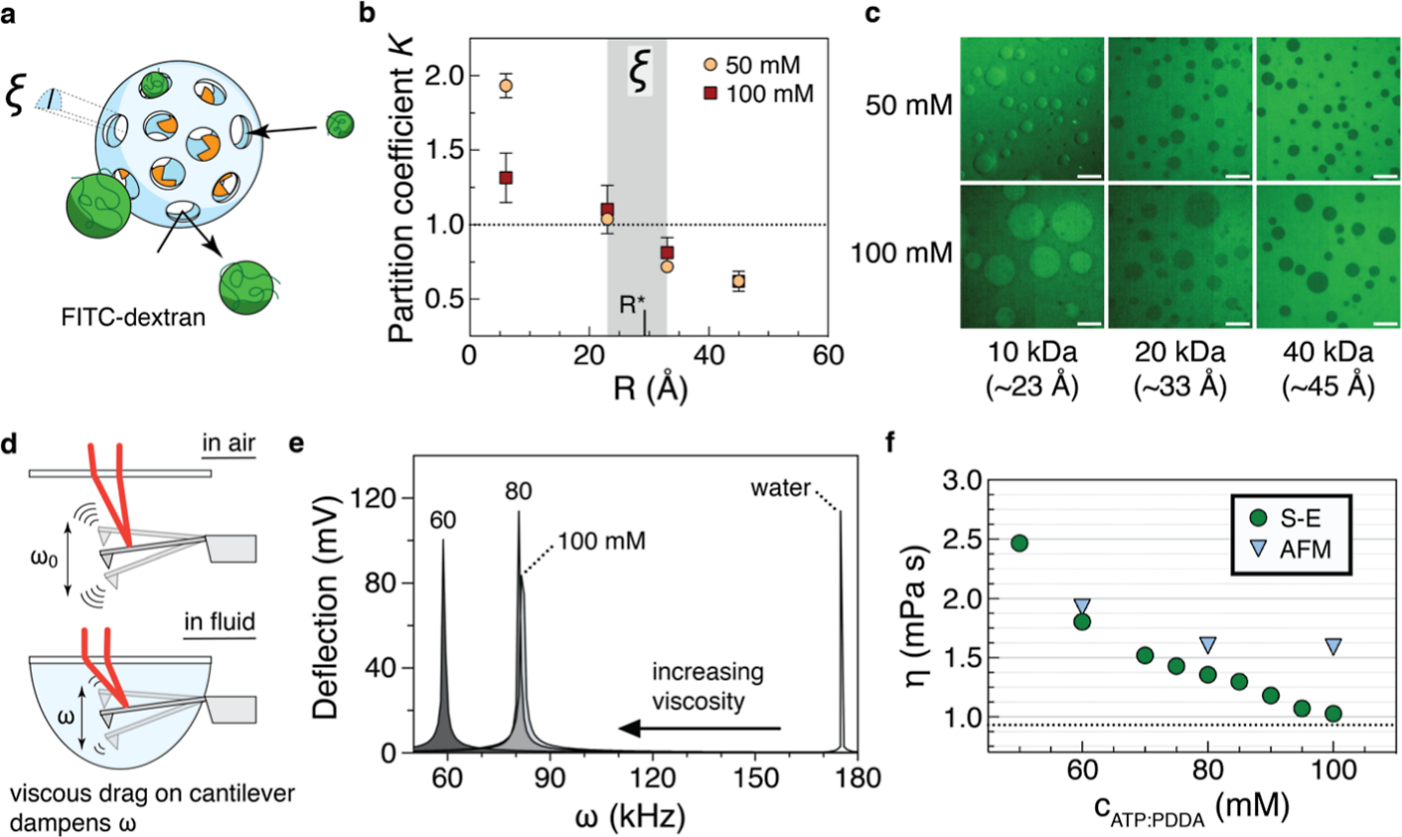
Material properties of the underlying ATP/PDDA coacervate. (a) Conceptual schematic demonstrating how partition experiments can estimate the coacervate mesh size ξ. If a coacervate droplet is imagined to be a wiffle ball with pores having radii ξ, then fluorescent probes like FITC-dextran will either enter the droplet (radii < ξ) or be excluded (radii > ξ). The radii of probes whose phase preference changes from solvent to coacervate phase would constitute a ξ range. (b) Partition coefficients of FITC-dextrans 10 K (23 Å), 20 K (33 Å), and 40 K (45 Å) in both 50 and 100 mM coacervates. Free Atto488 dye was used to show partitioning of a single nm-sized probe. The predicted coacervate mesh size is highlighted as a gray region between 2.3 and 3.3 nm; dextranase’s radius is marked within this region. Data shown as mean ± SD, *n* = 25–30 droplets. (c) Representative images showing FITC-dextran 10 K, 20 K, and 40 K partitioning in the two coacervate conditions; scale bars, 10 μm. (d) Cartoon showing principle of cantilever viscometry. Micron-scale cantilevers experience thermal fluctuations quantified as the resonant frequency ω. Upon immersion in fluid, ω dampens as the cantilever experiences viscous drag, with decreasing ω corresponding to increasing viscosity. (e) Resonance data for a cantilever immersed in 60, 80, and 100 mM ATP/PDDA coacervates and in water for comparison. Graphs represent the average of 11 measurements. (f) Droplet viscosity calculated from dextranase diffusion (green circles) and ω (blue triangles). Respective points were calculated from data in panel (1c) and from Gaussian fits to data in panel (e). S–E: Stokes–Einstein, AFM: atomic force microscopy.

Turning to the chemical composition of the coacervates, we considered how the coacervate phase volume grew (where the volume ratio tended to 1:1) with increasing ATP/PDDA concentrations. Given the difficulty of measuring the PDDA concentration in each phase, we used spectrophotometry to probe ATP concentrations. After measuring ATP concentrations in a range of enzyme-free coacervate preparations, we computed partition coefficients and plotted both sets of data against enzyme diffusion measurements subsequently performed with those coacervates (Figure S9a,b). In line with expectation, enzyme diffusion decreased as ATP partitioned more into the coacervate phase. What is notable here is that partitioning was inversely related to the initial ATP concentration, suggesting that ATP itself has some physical influence within the coacervate.

Investigating further, we noticed that for initial overall ATP concentrations at and above 80 mM, the concentration of ATP in the coacervate becomes lower than the initial ATP level. This decrease in the ATP concentration in the coacervate phase follows a near-linear decreasing trend (Figure S9c). Combined with the volume ratio observation above, this suggested that more water enters the coacervate at higher ATP/PDDA formulations. Furthermore, it was observed that solutions with less than 1 mM and more than 200 mM (up to 600 mM) ATP would not form a turbid mixture upon addition of PDDA. There may then be at least two critical points on the ATP concentration axis, such that the phase diagram for an ATP/PDDA coacervate is likely to have a closed-loop binodal curve^47^ (Figure S9d). These phenomena may be explained by how ATP contributes to a charge imbalance, at which certain excess or deficit of ATP leads to no observable LLPS. This is not specific to ATP itself but for any ion. Regarding the organelle viscosity, we note that polyelectrolytes tend to have chains collapse in the presence of counterions,^48^ strongly so when multivalent,^49,50^ and this structural change leads to a large decrease in viscosity.^49,51^ This has been shown for PDDA^52,53^ and other cationic polyelectrolytes^54^ when mixed with monovalent anions. We measured bulk viscosities for the coacervate at select concentrations using an AFM cantilever-based method^32,33^ and confirmed the drop in viscosity corresponding to increasing solution ATP (Figure 3d,e). Viscosities could also be calculated via Stokes–Einstein calculations from the dextranase diffusion data and were compared to the values from AFM (Figure 3f), showing reasonably close agreement. We may expect that ATP (or any ion) screening effects on PDDA and subsequent chain collapse lead to the observed lower bulk coacervate viscosity, thereby lessening the subdiffusive behavior of the enzyme.

### 3.4 Catalysis-Mediated Impact on Mesh Restrictions

Lastly, we sought to underscore the importance of phase-separated bodies by mechanistically describing what happens when there is no compartmentalization. To achieve this, we investigated how dextranase would diffuse differently in two polymer solutions, which were either inert (PEG) or sensitive (dextran) to dextranase activity. Our focus was first placed on the role the polymer regime may impart on enzyme diffusivity, i.e., on how dextranase might behave in concentrated polymer solutions as they approached and surpassed the overlap limit c*. Once the concentration of a polymer reaches c*, individual polymer molecules are statistically assured to be in contact and the solution is said to be semidilute.^45^ At and above this limit, the polymers form a mesh with an average distance of ξ between polymer blobs. Overlap concentrations using known values of PEG^55^ and dextran radii of gyration were calculated and used as a basis for these experiments. Using only the bulkier polymers PEG 400 kDa and dextran 500 kDa, we found that the dextranase diffusion trended downward as the polymer concentration approaches c* but trended even further downward as the polymer passes c* (Figure 4a). This trend appeared to be indifferent to whether dextranase could cleave or even bind to the polymer, thus suggesting that macromolecular crowding can have a negative impact on enzyme function in cases where mobility is directly tied to catalysis, as would be the case for diffusion-limited enzyme reactions (Figure 4b).

**Figure 4:**
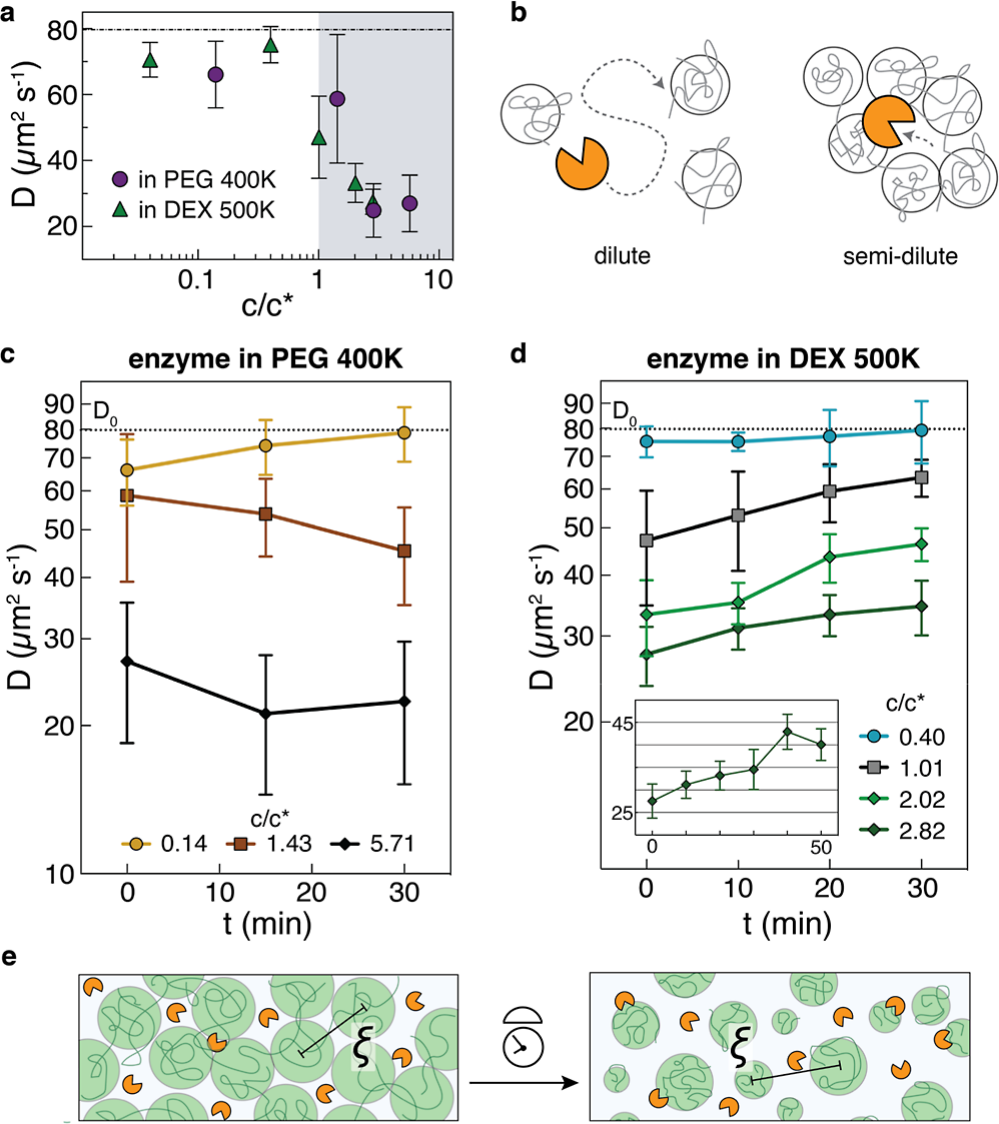
Space is important for dextranase when embedded in a crowded and viscous substrate environment. (a) Enzyme diffusion was measured immediately after adding dextranase to a range of concentrations of PEG 400 K and dextran 500 K, which are given as scaled to the calculated respective overlap concentration c*. There is an observed change in the diffusion trend as the polymers cross into the semidilute regime (*c* > c*). Data shown as mean ± CI, *n* = 10 measurements. (b) Cartoon showing dextranase has limited mobility when in a semidilute polymer solution, independent of whether the polymer is a substrate or not. (c) Dextranase diffusion within the first 30 min after injecting enzyme into solutions of varying PEG 400 K concentrations, relative to c*. Data shown as mean ± CI, *n* = 10 measurements. (d) Dextranase diffusion within the 30 min after injecting enzyme in varying concentrations relative to c* of dextran 500 K. The inset shows an extended time look of the 70 mg mL^–1^ (*c*/c* = 2.82) condition, where the enzyme continued to increase in diffusivity. Data shown as mean ± CI, *n* = 11–16 measurements. (e) Schematic depicting the growth in the mesh size ξ as enzyme actively increases local space for itself among a coterie of dextran blobs.

We note though that these FCS measurements were taken immediately upon addition of the enzyme to the polymer solution, so we next measured diffusion in 10 to 15 min increments after enzyme introduction. First, in PEG 400 K, to which dextranase should not be catalytically active, we did not observe any increases in diffusivity over a 30 min period, except in the dilute condition where diffusivity trended toward baseline but still within range of error (Figure 4c). Second, for dextran 500 K, the data showed that again, the enzyme has the lower initial diffusion as expected in the semidilute regime, but critically, in the dilute regime, dextranase remains near its normal freely diffusing state (Figures 4d and S10). We note that enzyme diffusivities can be seen as trending upward in all dextran conditions, with the means increasing the most in the case where *c*/c* ≈ 1. A slowdown in the most concentrated case (70 mg mL^–1^) is not surprising, but as shown in the inset, enzyme mobility continued to increase beyond the initial observation period, with means reaching as high as nearly 1.7 times the initial diffusion. This is relevant in the context of enhanced diffusion; as dextranase encounters growing amounts of small dextran fragments, such increases in diffusion are consistent with previous reports examining catalyzing enzymes.^56,57^ However, reports conflict over the basis of such apparent diffusivity increases,^58^ though techniques like single-particle tracking in polymer solutions^59^ support the notion of anomalous diffusion. We performed scanning STED-FCS experiments to further investigate the diffusive heterogeneity in our reactive system, the results and further discussion of which can be found in Figure S11.

The mesh size ξ typically decreases as polymer concentration increases, and at a *c*/c* of 1, ξ is approximately equal to the polymer’s radius of gyration *R*_g_.^60^ In the case of dextran 500 K, the *R*_g_ is about 20 nm. Thus, in the result above for *c*/c* ≈ 1, we might expect that the initial low diffusion is due to substrate docking rather than complete trapping. We rationalize substrate docking by how in the results for PEG 400 K, which have similar radii, there were several data points near *D*_0_, suggesting the enzyme was either free or transiently trapped within the PEG mesh; there are far less measurements in dextran near *D*_0_. Given that at higher concentrations ξ approches the enzyme’s radius, we interpret the higher concentration results—which had noticeable increases in diffusion over time—to indicate that dextranase frees itself from dextran’s mesh restriction as it acts to hydrolyze dextran (Figure 4e). The increase in diffusion corresponding to a likely increase in ξ suggested to us that some enzymes may operate ideally only when compartmentalized away from a highly viscous or bulky substrate, contrary to the general belief that enzymes must be colocalized with their substrates to be effective.

## 4 Conclusions

Conventional explanations of condensate-mediated catalytic enhancement rely on colocalization of enzyme and substrate to increase effective concentrations. Based on this theory, smaller condensates would be desirable for increasing reaction rates as they maximize enzyme concentration. Yet in cells, increased enzymatic activity may coincide with increased condensate size. This might therefore apply in early life situations, with the first protocells formed via phase separation. Under what circumstances might larger condensates or organelles lead to increased catalytic efficiency, given that larger compartments may decrease effective concentrations of partitioned molecules? Here, we demonstrate that a proto-organelle composed of 1:1 ATP/PDDA displays larger coacervate volume fractions when increasing initial concentrations. Importantly, the higher volume fractions correlate with the enhanced reaction efficiency as physical changes within the droplet permit recovery of enzyme diffusivity, enabling more frequent interactions between the compartmented enzyme and the excluded substrate. The simplicity of our system could inform rapid fitness tests for the first proto-organelles, where lower viscosity proto-organelles would immediately gain an evolutionary advantage.

Previous work^8,61–63^ helped uncover that higher effective concentration is an important underlying factor behind some forms of condensate-mediated enhanced enzymatic activity. However, we should note that if higher concentrations are achieved through decreasing compartment size by way of increasing external/internal phase volume ratio, then in the context of control theory, such a strategy is a fragile control method. In this manner, maximal enzyme activity is reached when approaching the phase boundary (Figure S12a), where system stability is susceptible to fluctuations and cannot be easily modeled,^64^ especially in the case when the polymer concentration is very low. One might expect the same instability when considering coacervate viscosity in that the lowest viscosity enabling maximum enzyme reaction rates would be found near the phase boundary. However, in this work, generating coacervates within a wide concentration range of initial ATP and PDDA gives sliding control over dextranase activity by gradually transitioning from a well-mixed regime to small, highly viscous condensates to larger, low viscosity condensates. This type of system of enzyme reaction rate control is robust as the increasing polyelectrolyte concentration promotes stability by further moving the system within the LLPS region of the phase diagram and away from critical zones (Figure S12b). Yet, a major caveat is that enzymes would need to be diffusion-limited for this method to be feasible. Work until now has established that enzyme *k*_cat_ or apparent substrate *K*_m_ can be changed, such as by controlling partitioning of regulators that affect polymerase activity,^65^ and this does constitute a significant and viable method for robust control within cells, giving support to the strategy of increasing effective concentration. Control over partitioning has been shown to be facilitated by high (∼Pa s) droplet viscosity,^16,66^ and our findings complement those studies and the above concepts by highlighting (diffusion-limited) enzyme mobility as an alternative robust regulatory control point.

The work presented here also leads to new questions, such as if diffusion-limited enzymes in the cell localize to specific droplets to maximize catalysis of condensate-excluded substrates or simply avoid inhibition by large, bulky substrates. Additionally, the diffusion-based robust control principle outlined above could apply to different types of nuclear condensates,^2^ where transcription factors and polymerases (which appear to be at or near the diffusion-limit^67^) localize to specific condensates that are hotspots for transcriptional activity.^68^ Slow diffusion resulting from crowding also appears to be a problem for translation.^69^ The specific model presented in this paper, in which an enzyme diffuses within a droplet of lower viscosity than the surrounding polymeric substrate, also parallels the nucleolus, which has significantly lower viscosity^70^ than the chromatin-rich nucleus^71,72^ (Figure 5a). In consideration of the physical properties that may affect the substrate-excluded enzymatic rate constant *k*, we make use of the mutual diffusion coefficient $D_{m}=D_{enz}+D_{sub}\approx {\left(\eta_{org}+\eta_{sub}\right)}^{-1}$ (ref 73). The general Smoluchowski equation for diffusion-controlled reactions calculates *k* = 4π­(*r*_enz_ + *r*_sub_)*D*_m_, in which the enzyme and substrate radii affect the enzyme reaction rate. In our case of substrate-exclusion, surface area must also be accounted for in the equation, and given that intuitively, surface area (*k* ∝ *r* ^–2^) counters diffusion (*k* ∝ *r* ^2^), we arrive at a simplified, viscosity-dependent rate constant approximated as $k_{\eta }∼{\left(\eta_{org}+\eta_{sub}\right)}^{-1}$ , in which the viscosities of the condensate and the substrate significantly impact enzyme turnover. We plot the results of this equation for a range of viscosities relevant to our system in Figure 5b. In the case of substrate-including organelles, we expect η_sub_ ≈ η_org_, such that for these organelles/condensates, they are placed along the angled dashed line in the plot. We note that the predicted activity increases, of nearly 2 orders of magnitude, is in conjunction with the slope differences observed in Figure 2b. Returning to the degradation data, we fitted the well-known Gompertz function,^74–76^ $\mu =e^{-be^{-rt}}$ , where *b* is a factor affecting the initial degradation rate and *r* is the extent of reaction acceleration (Figure 5c, left). We found that *b* values change slightly, in correspondence to the relatively small initial contributions η_org_ has for *k*_η_ given η_org_ ≪ η_sub_. However, *r* changes dramatically, increasing from −0.034 in the 25 mM coacervate to −0.247 in the lower-viscosity 100 mM coacervate (fold changes are shown in Figure 5c, right). This change in the measure of reaction acceleration is consistent with the increasing impact of η_org_ as η_sub_ decreases due to substrate degradation. Both *b* and *r* can be linked to the arrows in Figure 5b: *b* corresponds to the downward arrow, where a viscosity change via compartmentalization (and alleviation of substrate inhibition) greenlights the reaction acceleration seen in the leftward-pointing arrow; *r* accounts for the rate at which the leftward arrow accelerates toward the *y*-axis.

**Figure 5:**
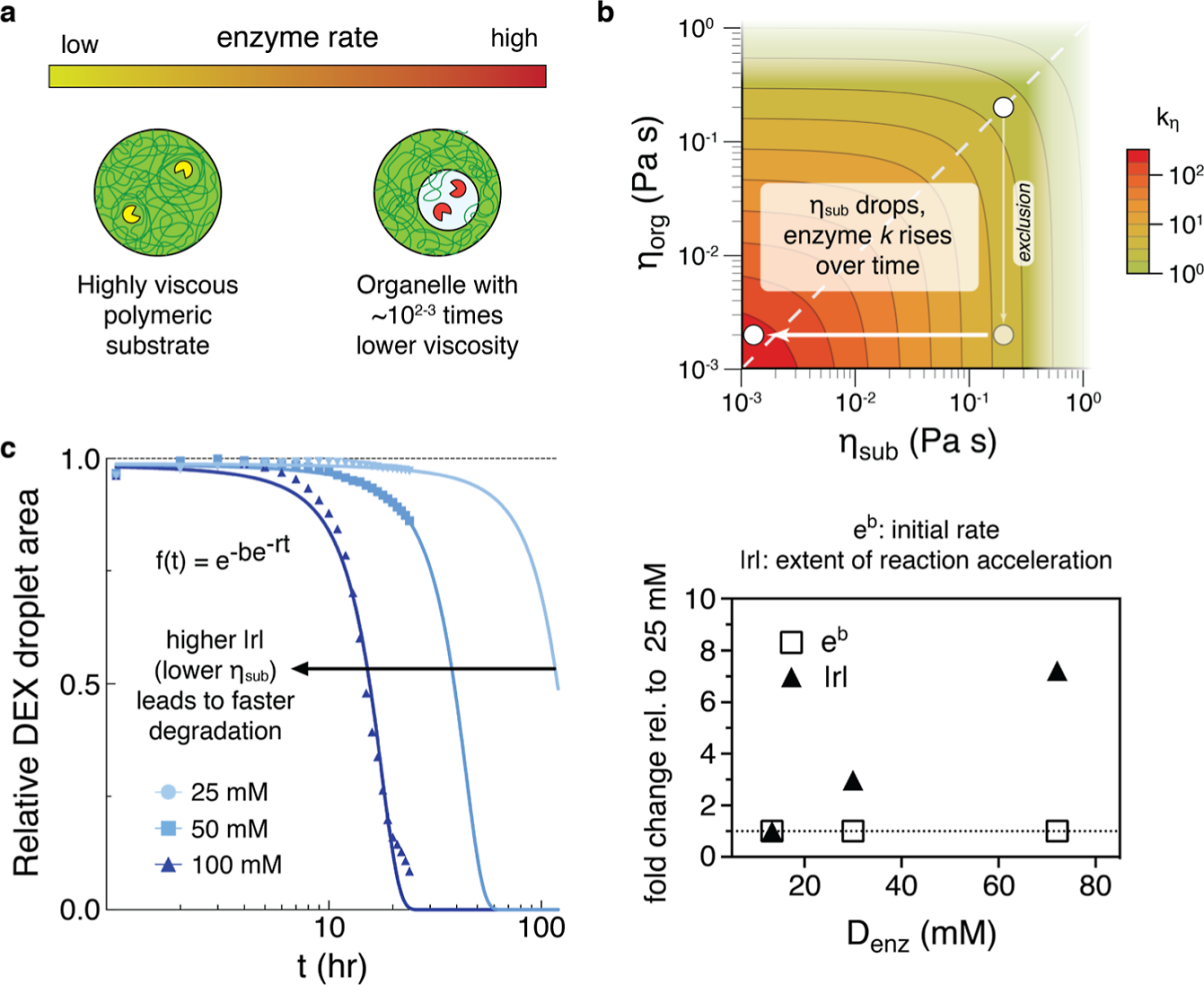
Strategies for adaptation to a dense polymer environment. (a) Schematic showing the utility of substrate exclusion. By ejecting a highly viscous polymeric substrate, a lower viscosity condensate or organelle can exhibit higher enzyme activity. (b) For an enzyme with diffusion-controlled characteristics, effects of displacement (*k* ∝ *r* ^2^) and droplet surface area (*k* ∝ *r* ^–2^) cancel out in contributions to the rate constant. A viscosity-dependent rate constant can then be formulated as $k_{\eta }∼{\left(\eta_{org}+\eta_{sub}\right)}^{-1}$ and a range of viscosities produce the contour plot shown. The angled dashed line corresponds to existing models, in which enzymes and substrates are colocalized. Compartmentalization overcomes the initial barrier imposed by substrate viscosity, unleashing the enzyme and allowing for accelerating enzyme activity as the substrate undergoes depolymerization. The change in activity nears 2 orders of magnitude, achieved with the substrate in an initial concentrated regime. (c) The degradation data from Figure 2 is fitted to the Gompertz function (shown as a function of time; left panel). There are two parameters from fitting, *b* and *r*, which correspond to the coacervate viscosity effects on the initial rate and reaction acceleration, respectively. While *b* increases only very slightly as coacervate viscosity is lowered, *r* increases significantly (right panel), leading to accelerating degradation times.

Autocatalytic reactions are believed to play an important role in origin of life systems.^77^ Our proto-organelles attempt to lay groundwork for future realization of such postulated autocatalytic features.^78^ We hypothesize that proto-organelles could achieve accelerating, physical autocatalysis reaction networks simply by excluding the substrate. Lower viscosity driven by concentrating the phase-forming components, as possible by evaporation in prebiotic life,^79^ would accelerate physical autocatalysis. Autocatalysis has been observed previously for hydrolytic reactions but are typically dependent on chemical properties, such as pH,^80^ polymer size and structure,^81,82^ and ionic strength.^83,84^ Autocatalysis in this work is achieved physically as substrate exclusion is the induction step before enzymatic hydrolysis of the substrate leads to lower substrate viscosity and subsequently autoacceleration. The work here provides a context for evolutionary fitness, in that differing viscosities could create a diverse population of constitutively or transiently active organelles; specifically, particular protocells could survive where organellar activity can be tuned individually and/or balanced by differently active organelles, offering the cell an ability to navigate its environment. In general, our approach suggests improving *k*_cat_ or *K*_m_ by concentration enhancement^65^ may not be enough to explain the effectiveness of compartmentalization for enzyme activity; diffusion must be considered as well.

To expand upon this work to a much broader context, our studies of dextranase may also find direct application in industries ranging from food to healthcare. Dextranase is used not only to remove large dextrans that limit sugar yields but also to process dextran into forms useful for cosmetics and food stabilizers.^85^ Additionally, dextran is a critical component of dental plaques^86^ and enzyme-based treatments are desired though difficult to achieve. The work presented here on dextranase could ameliorate the effectiveness of the enzyme in these applications. Further work involving substrate-excluding phase-separated droplets could provide fundamental insight into the function and regulation of protocells, cellular condensates, and myriad cellular processes involving hydrolytic or diffusion-limited enzymes.

## Supplementary Material



## Data Availability

The data that support the findings of this work are available from the corresponding author, S.T., upon reasonable request.

## Notes

Analysis of dextranase *k*_cat_/*K*_m_, dye effect on partitioning behavior of labeled dextranase, diffusion measurement of free dextranase in buffer, time-course measurement of dextranase partitioning in coacervates, internal phase volume of different coacervate formulations, coacervate droplet dewetting behavior, nanorheological probing of coacervates, zeta-potential measurements of fluorescent dextrans, ATP concentration measurements and comparison to enzyme diffusion, examples of raw, non-normalized FCS data and fitting, scanning STED-FCS measurements and heterogeneity analysis of dextranase in dextran solutions, and control theory context of the coacervate system used in this work (PDF)
