# Supplementary material for: Substrate Exclusion Greenlights Physical Autocatalysis of Enzyme Activity in Membraneless Proto-Organelles
Source: Biomacromolecules. 2025 Oct 29;26(11):7398–409. doi: 10.1021/acs.biomac.5c00926 (PMC12606559; doi:10.1021/acs.biomac.5c00926)
Supplement: Supplementary file 1 [file bm5c00926_si_001.pdf]

Supplementary Information for **Substrate exclusion greenlights physical autocatalysis of enzyme activity in membraneless proto-organelles**

Tasdiq Ahmed[1], Adya Verma[1], Shuichi Takayama[1,2,*]
1. Wallace H Coulter Department of Biomedical Engineering, Georgia Institute of Technology and Emory University, Atlanta, Georgia 30332, United States
2. Petit Institute for Bioengineering and Bioscience, Georgia Institute of Technology, Atlanta, Georgia 30332, United States
*Correspondence: takayama@gatech.edu

Supplementary Methods and Discussion

ATP concentration measurements 3

Contact angle measurements 3

Nanorheology 3

Zeta potential measurements 3

Scanning STED-FCS 4

Supplementary Figures

1. Dextranase may be a diffusion-limited enzyme for dextran 500 kDa. 5

2. Labeled enzyme localization can be affected by choice of dye. 6

3. Establishing baseline diffusion of dextranase in buffer. 7

4. Dextranase is largely confined to the coacervate even under the surrounding dextran environment. 8

5. Increasing ATP:PDDA concentration corresponds to increasing coacervate phase volumes. 9

6. High concentration coacervate droplets exhibit dewetting behavior on hydrophilic surfaces. 10

7. Initial nanorheological probing suggests the coacervate mesh size is near dextranase's radius. 11

8. Zeta potential measurements suggest fluorescent dextran partitioning is not significantly influenced by charge. 12

9. Further linking coacervate composition and enzyme diffusion. 13

10. Example of raw correlation data and fits in the active enzyme state. 14

11. Scanning STED-FCS uncovers heterogeneity in active dextranase. 15

12. Increasing concentration is a robust control alternative to the established fragile method of increasing volume ratio and partitioning. 17

Supplementary References 18

Supplementary Methods and Discussion

ATP concentration measurements

The concentration of ATP inside the coacervates and in the external phase were measured using a NanoDrop spectrophotometer (Thermo Fisher). Samples were diluted 1000-2000x before measurement, and the dilution factor was accounted for in calculations. ATP strongly absorbs light at 260 nm with an extinction coefficient of 15.4 mM^{-1} cm^{-1}. PDDA did not absorb light in the tested range. Absorbance values were corrected for background.

Contact angle measurement

Turbid solutions of 100 mM ATP:PDDA coacervates loaded with µg mL^{-1} amounts of Atto488-COOH dye were pipetted into the wells of an 8-well high glass bottom slide (ibidi) coated with 1% Pluronic-F127 (see main text methods for slide preparation). Z-stack images were collected using a Nikon W1 spinning disk confocal microscope with a step size of 0.2 µm. The stacks were imported into Volocity and rendered in 3D, adjusted for brightness/contrast, and cross-sectional images of droplets were exported. Contact angles were measured using ImageJ.

Nanorheology

To probe the mesh size of the coacervates, two experiments were performed. The first, as in this supplementary document, was by measuring the diffusion of fluorescent polysaccharides within a coacervate. Dextrans and Ficoll are considered spherical with well-known approximate hydrodynamic radii. We inject low concentrations (1-10 µg mL^{-1}) of FITC-dextrans (20k, 40k, 500k), FITC-Ficoll (400k), and free Atto488 dye into a 2.5 mM ATP:PDDA coacervate. By FCS, diffusion coefficients were measured and using the spherical approximation and the Stokes-Einstein relation, the viscosity experienced by the probe could be calculated.

Zeta potential measurements

The zeta potential of FITC-dextrans at 25˚C were measured using a Zetasizer Nano Z (Malvern Panalytical). Each fluorescent dextran was prepared in distilled water at ~1 mg mL^{-1} and diluted down ten-fold for the experiment. Potassium chloride (final concentration of 10 mM) was added to each solution to suppress background noise during measurement.

Scanning STED-FCS

Experiments were performed on an Abberior Facility Line microscope as used in the main text. STED laser powers were first measured using a power meter (ThorLabs) and calibrated against 20 nm fluorescent beads (FluoSpheres, Invitrogen) to generate a record of beam waists for scanning STED-FCS experiments. For the experiment, samples were prepared exactly as for the regular FCS experiments in the main text, except with the addition of 80% glycerol. Such a high concentration of glycerol was needed to slow down the enzyme enough to be caught by the instrument scanner. Orbital scans were collected through Imspector software (Abberior) with frequency of 4.98 kHz, radius 2 µm, pixel size 10 nm, and dwell time 1 µs. The resulting intensity carpets were imported into the open source FoCuS-scan software[1] to generate correlation carpets; carpets had spatial binning applied to reduce noise, but no photobleaching corrections were used. Individual curves of the carpets were exported and fitted in MATLAB using the well-defined equation for scanning FCS[2,3]. Data was then processed in Microsoft Excel and Graphpad Prism.

Analysis of all data followed the workflow established in ref. 2. First, diffusion coefficients for the various STED powers were plotted against calculated observation volumes, to discern if a pattern formed. If for example diffusion coefficients were mostly similar in range across the different volumes, then dextranase is freely diffusing. As can be seen in Suppl. Fig. 11, dextranase exhibited decreasing diffusion as focal volume shrunk, indicating the enzyme was hindered. This corroborated the point FCS results in the main text Fig. 4. However, further analysis via Gaussian fitting of the individual transit times, of which nearly 200 datapoints were available, revealed the enzyme could be split into main and tail components. The tail component transits are slower diffusing and are anticipated as being substrate-bound. The main component transits are Gaussian conforming and likely a mix of bound and free enzyme. In the first 30 minutes of reaction, the tail transits compose as high as ~37% of total transits, reflecting high activity and substrate docking. While this is straightforward, a closer look at the main component reveals a shift towards lower diffusion times, which can be seen in the STED histograms. Comparison of the ratio between STED and confocal diffusivities confirms that as the reaction progresses, dextranase appears to recover to a freely diffusing state, despite the presence of glycerol.

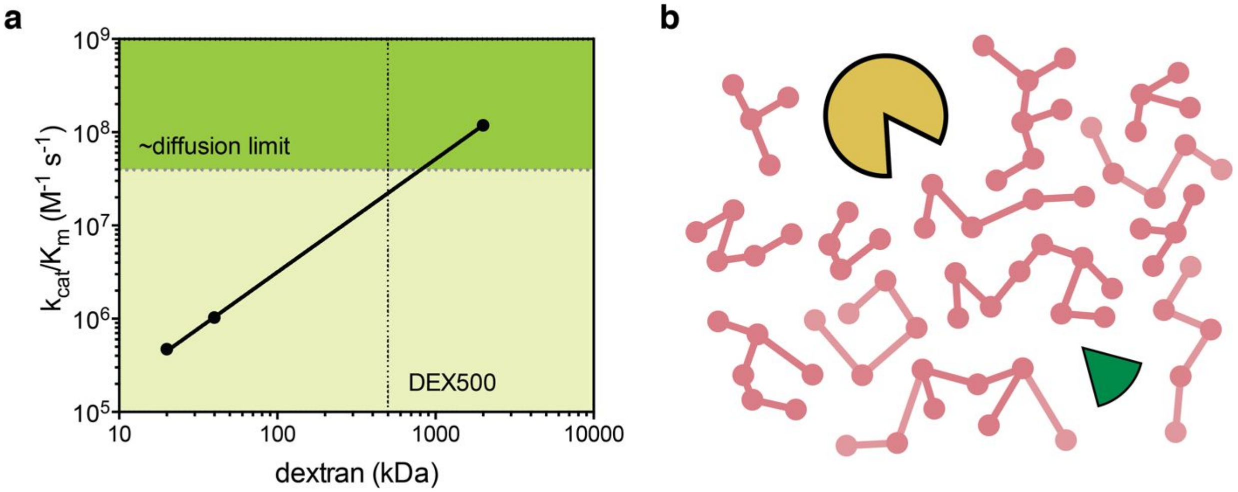

Supplementary Figure 1. Dextranase may be a diffusion-limited enzyme for dextran 500 kDa. (a) For high molecular weight dextrans, dextranase is quantitatively a diffusion-limited enzyme. We calculated the k_{cat}/K_m ratio for an endodextranase using published values[4] and plotted them against the dextran molecular weight, applying a log-log fit to approximate enzyme performance across the mass range. A diffusion limit of 4×10^7 was set based on a list[5] of these so-called "catalytically perfect" enzymes. For dextran 500K (the substrate we use for dextranase assays), the predicted ratio is ~ 2.2×10^7, which is reasonably near the diffusion limit. However, the numbers used in this analysis are for a specific, isolated strain of dextranase. Though we cannot verify the accuracy of these numbers for the commercial-source enzyme we use, we note that dextranases have long been known to exhibit the same trend of increasing k_{cat}/K_m ratio[6-8]. This phenomenon does give reason to study further the effect increasing diffusion may have for dextranase, even if not the ideal diffusion-limited enzyme. (b) General schematic for a diffusion-limited enzyme. Here, the enzyme (gold) is physically blocked by the surrounding environment (crowders in red) from reaching its substrate (green) in the fastest time possible.

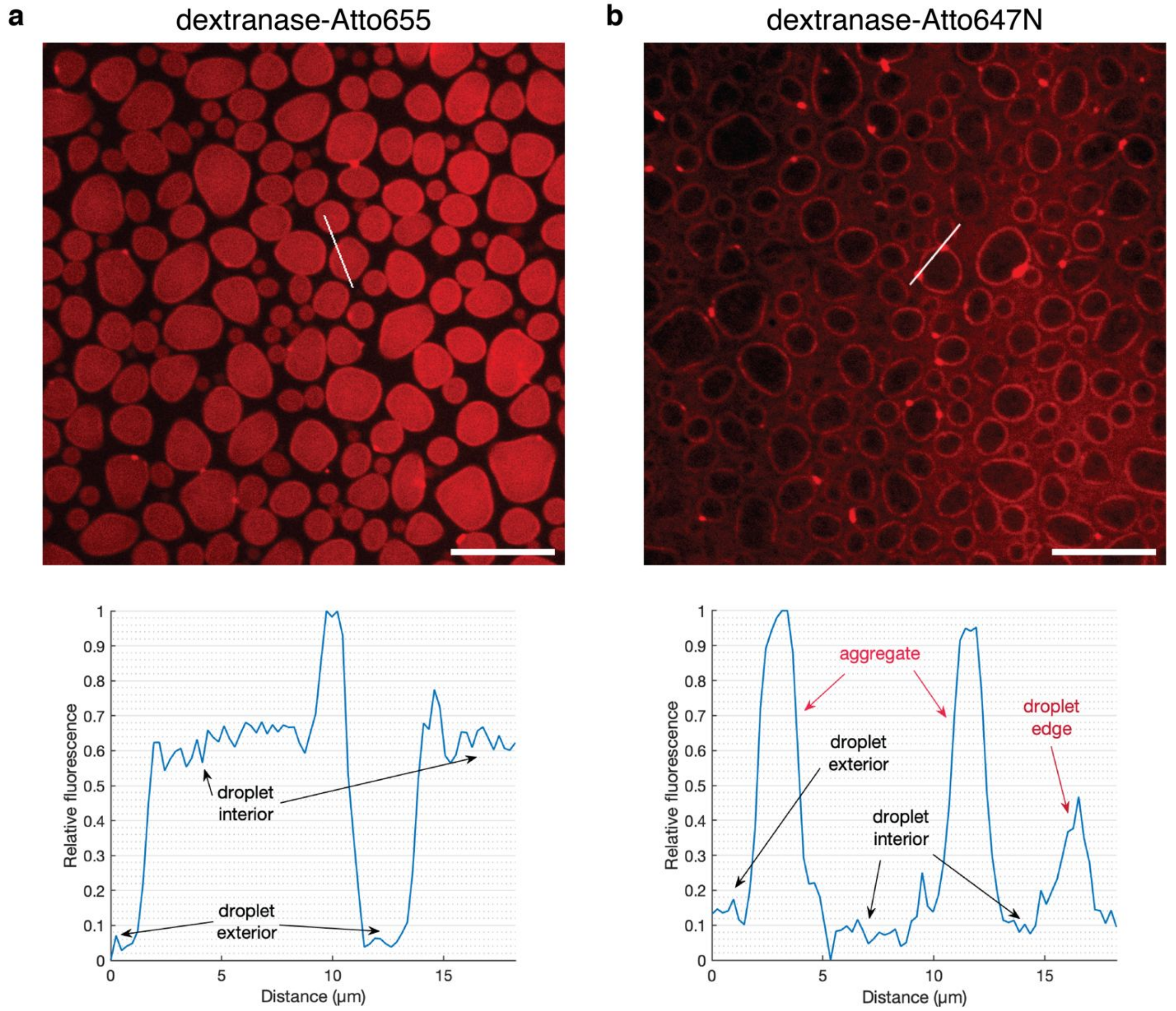

Supplementary Figure 2. Labeled enzyme localization can be affected by choice of dye. A commercially available dextranase was individually-labeled with one of two different dyes, Atto655 and Atto647N. Using the freely available logD plugin from MarvinSketch, we examined the red dyes Atto655 and the Abberior dyes STAR RED, STAR 635 and STAR 635P for their hydrophobicity at pH 6 and found that only the zwitterionic Atto655 dye has a sufficiently negative logD (logD < 0 implies hydrophilicity) value at pH 6, which gave us reason to explore Atto655 as an alternative to the benchmark Atto647N. (a) Dextranase-Atto655 can be seen as generally distributed throughout a 2.5 mM ATP:PDDA coacervate. (b) Dextranase labeled with Atto647N showed a strong localization to the coacervate-dilute phase interface. Line profiles show fluorescence increase at the droplet edge vs. interior. In both cases, protein aggregates could be observed but was more pronounced in the Atto647N case. Note that the result here does not confirm the distribution of non-labeled dextranase. Scale bars, 25 µm.

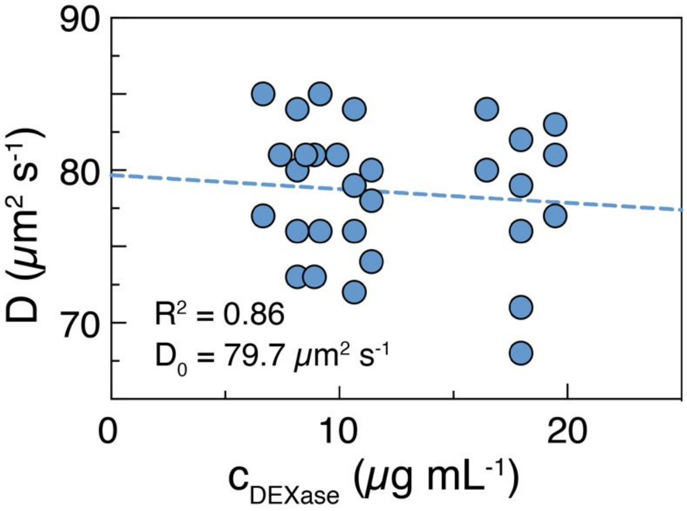

Supplementary Figure 3. Establishing baseline diffusion of dextranase in buffer. Diffusion data from FCS performed on dextranase-Atto655 in pH 6 MES buffer at three serially diluted concentrations (n=10 measurements each). The data was linearly extrapolated to infinite dilution, giving the enzyme diffusion coefficient to be 79.7 $\mu m^2/s$ at 23˚C, which is within reason of an enzyme with nominal weight 66 kDa. Using Stokes-Einstein and assuming the enzyme to have the specified weight, we estimate the enzyme hydrodynamic radius to be 2.92 nm.

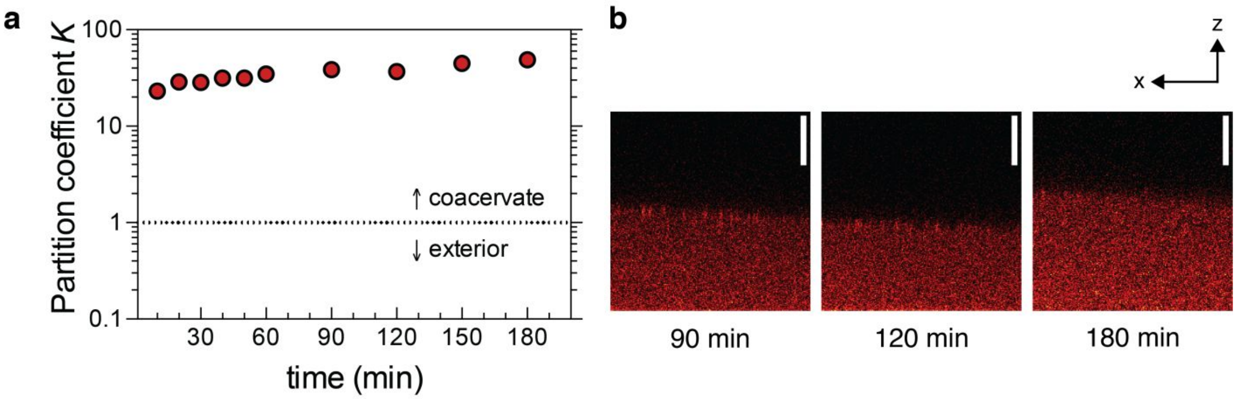

Supplementary Figure 4. Dextranase is largely confined to the coacervate even under the surrounding dextran environment. (a) Partition coefficients of dextranase-Atto655 in a 100 mM ATP-PDDA coacervate over time. A single coacervate pellet containing the enzyme was injected into a dilute solution of dextran 500K and images were collected over the course of three hours after addition. Values are calculated using intensities inside and just outside the droplet and were not corrected against background. (b) Cropped sections of x-z slices of the coacervate at 1.5, 2 and 3 hours after injection into dextran, showing droplet retention of enzyme. Note that the sections are not of the same area of the droplet.

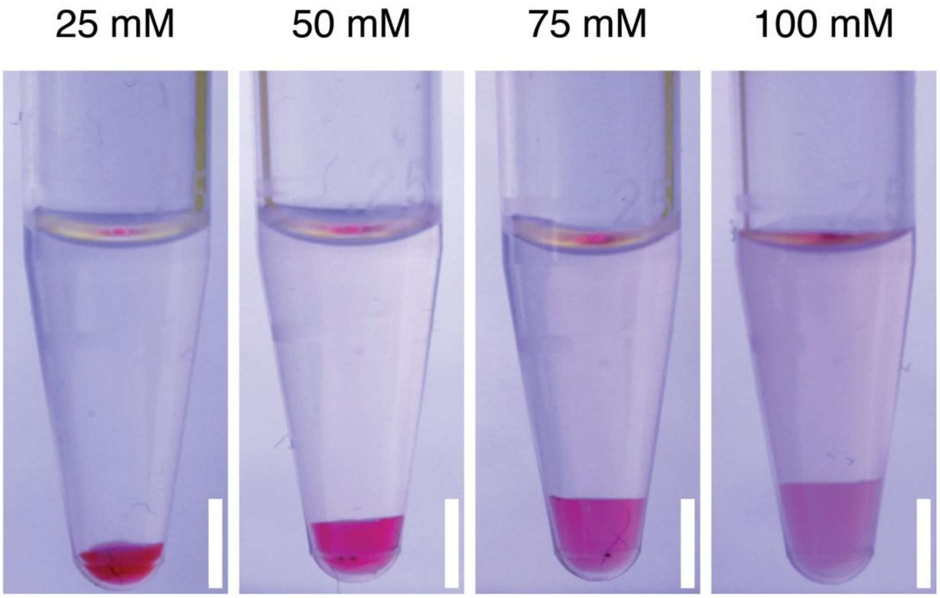

Supplementary Figure 5. Increasing ATP:PDDA concentration corresponds to increasing coacervate phase volumes. Drops of red food dye (which partition into the coacervate) were added to 1 mL of distilled water, mixed, and were added at further 100x dilution to turbid coacervate mixtures. All mixtures were prepared in a 500 µL Eppendorf tube and centrifuged for 15 min at 4000 rpm, 4˚C. Photos were taken in a low light area with a Canon EOS Rebel T6i at f/5.6, ISO 200 on a 52 mm lens with 1/30 exposure time. Images were edited for brightness/contrast in ImageJ. Scale bars, 4 mm. The pH for all conditions were observed to be similar, after testing the mixtures prepared similarly as above but without food dye.

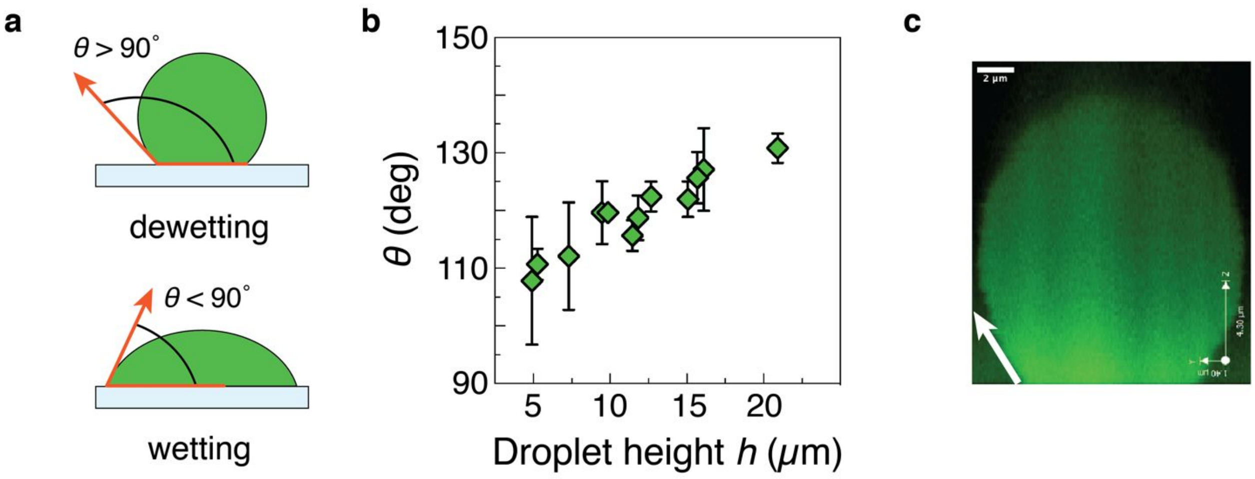

Supplementary Figure 6. High concentration coacervate droplets exhibit dewetting behavior on hydrophilic surfaces. (a) Schematic describing how measurement of contact angle can inform of wetting behavior. (b) Contact angles for 100 mM ATP:PDDA droplets measured on glass coated with 1% Pluronic-F127 (n=12 droplets, mean ± SD represents the contact angles measured on the central x-z and y-z slices for each droplet). (c) Example image of a 100 mM ATP:PDDA coacervate droplet containing Atto488 dye. Arrow highlights the obtuse angle associated with dewetting behavior.

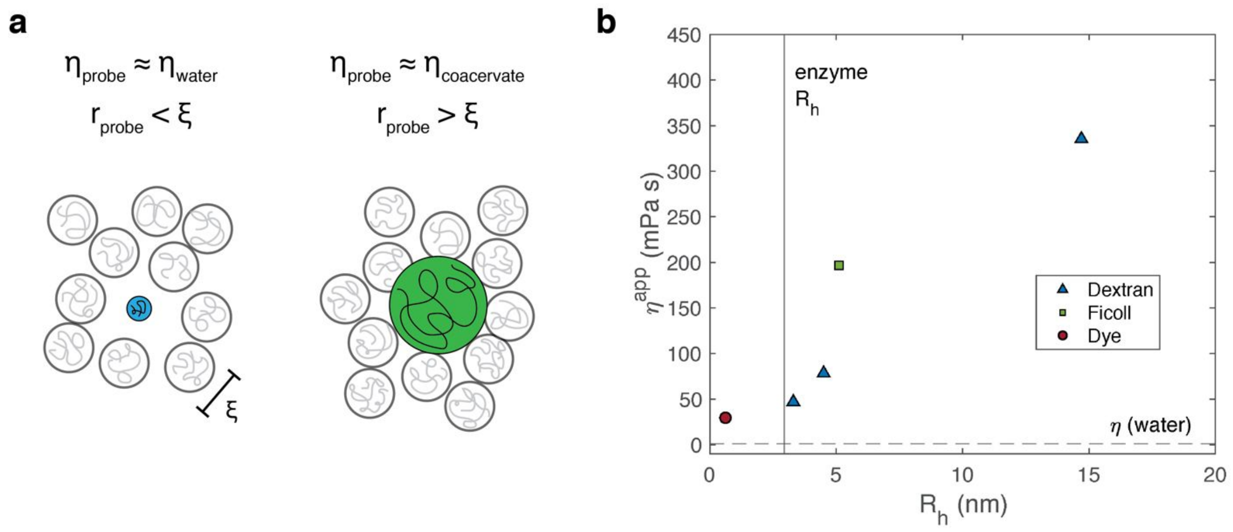

Supplementary Figure 7. Initial nanorheological probing suggests the coacervate mesh size is near dextranase's radius. (a) Schematic showing the concept of nanorheological probing to determine the polymeric mesh size ξ. Molecules with a radius below ξ will be immediately surrounded by water and thus experience a local viscosity approximately that of water, but molecules with a radius above ξ will instead experience a local viscosity similar to that of the polymer solution's bulk viscosity[9]. One could then estimate ξ with a series of probes containing radii both above and below ξ. (b) Various FITC-labeled probes known to be spherical (with the exception of Atto488-COOH dye which is a small molecule) were measured for their diffusion via FCS within a 2.5 mM ATP-PDDA coacervate. The apparent viscosities were calculated using Stokes-Einstein, inputting the single FCS result. The enzyme hydrodynamic radius is marked on the plot and was calculated from the diffusion coefficient in Supplementary Figure 3. The mesh size of the coacervate is expected to be within range of the enzyme, but a lack of single nm-sized probes makes mesh size determination in this manner difficult.

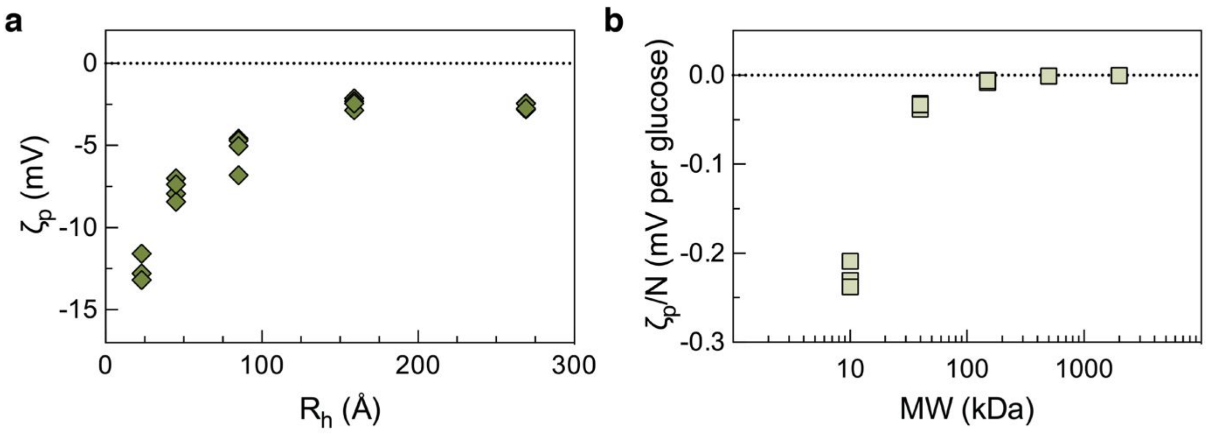

Supplementary Figure 8. Zeta potential measurements suggest fluorescent dextran partitioning is not significantly influenced by charge. (a) Zeta potential for various FITC-dextrans. Data for n=4 measurements on the same sample. (b) Zeta potential for the FITC-dextrans but normalized against the degree of polymerization. On average, a single dextran monomer is expected to have near zero charge, supposing all monomers contribute to the surface potential.

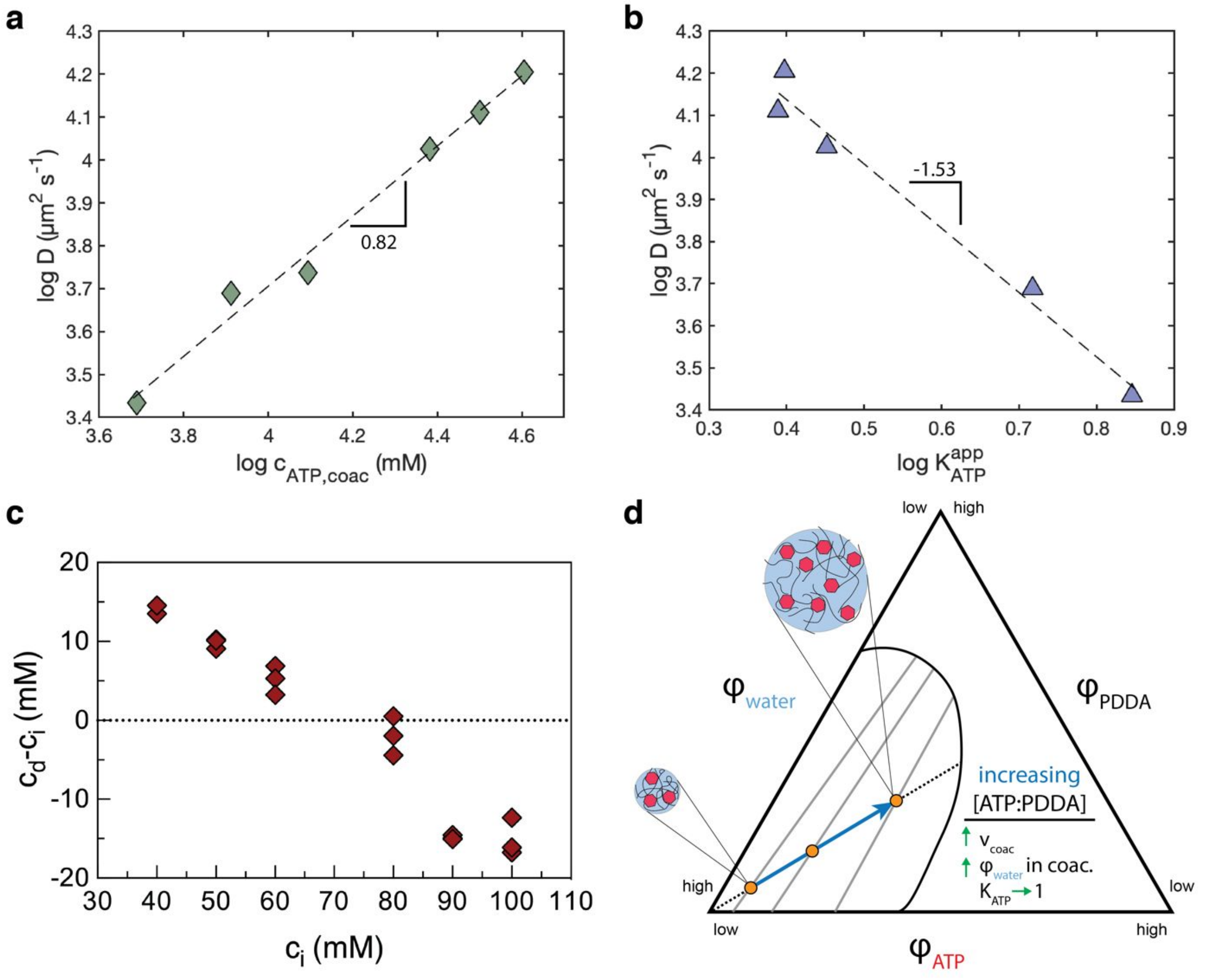

Supplementary Figure 9. Further linking coacervate composition and enzyme diffusion. (a) Log-log plot of enzyme diffusion vs measured ATP concentration in the coacervate phase. Scaling exponent in this case found to be 0.82. Diffusion data comes from an independent FCS experiment and ATP concentrations were measured using the same samples. (b) Log-log plot of enzyme diffusion vs ATP partition coefficient (defined as interior phase over exterior phase concentration). Scaling exponent found to be -1.53. (c) Residuals plot mapping the difference between the final coacervate dense [d] phase ATP concentration and the initial [i] pre-mixed ATP concentrations. Error bars represent standard deviation in the coacervate phase concentration (n=3 measurements). (d) Zoomed-in picture of a theoretical ternary plot highlighting two key features: 1. The tie line slope becomes more parallel to the water axis as one moves higher up the ATP volume fraction axis, indicating water-richness in the coacervate phase at higher starting compositions. 2. The binodal is likely to form a closed-loop, because concentrations above ~200 mM and below ~1 mM do not form coacervates. For easier visualization of the concentration trend used in our experiments, the binodal is cropped on the lower solute volume fraction ends.

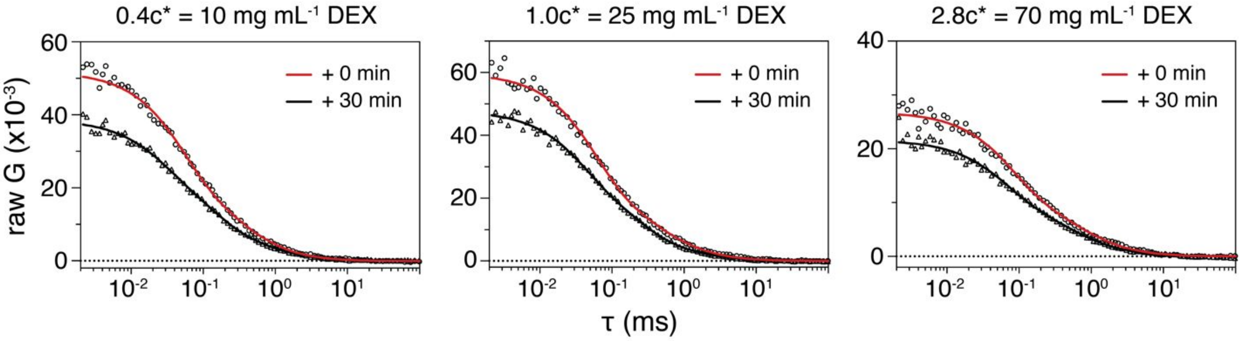

Supplementary Figure 10. Example of raw correlation data and fits in the active enzyme state. In the presence of dextran 500 kDa (concentration 10, 25 and 70 mg mL^{-1}), FCS data was collected for dextranase-Atto655 every 10 minutes following substrate introduction. Shown here are data points and resulting fit immediately following substrate addition (+0 min) and 30 minutes later. Note that the curves exhibit a traditional shape, suggesting traditional FCS fitting models are appropriate.

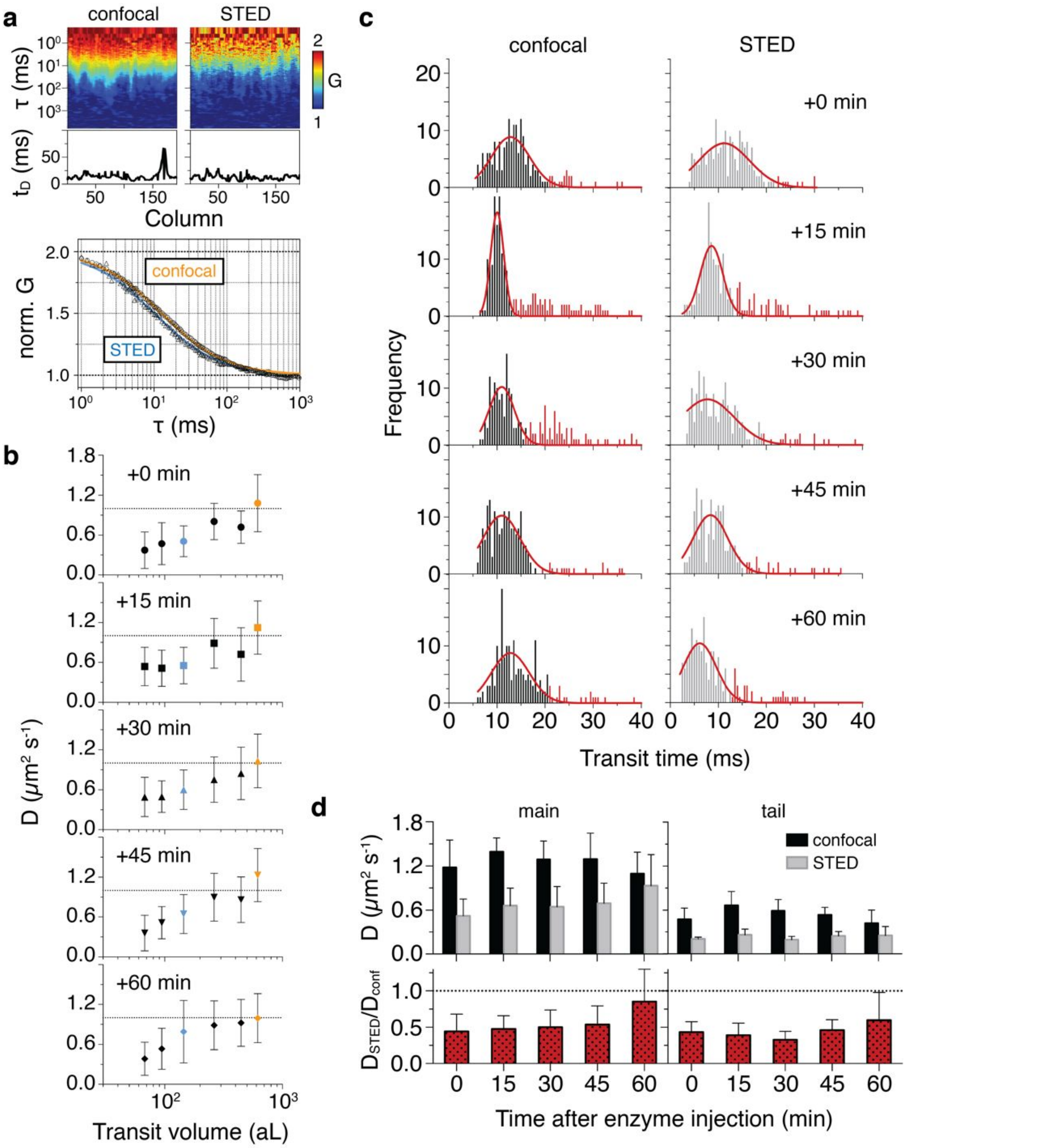

Supplementary Figure 11. Scanning STED-FCS uncovers heterogeneity in active dextranase. (a) Example confocal (vol~617 aL) and STED (vol~145 aL) correlation carpets immediately after introduction of 10 mg mL^{-1} dextran 500K into a solution of ~10 nM dextranase-Atto655 mixed with 80% glycerol. For each carpet, transit time t_D fits for each curve (N=192) are presented. Correlation and fit averages are plotted below. (b) Diffusion data from scanning measurements during 1 hr of enzyme-substrate reaction. Diffusion coefficients are plotted against observation volumes dependent on STED intensity. Increasing STED laser power decreases transit volumes and the observed drop in diffusivity indicates the enzyme experiences hindered diffusion in the reaction condition. The points used in panels c-d are highlighted in orange (confocal) and blue (STED). The two left STED points were not used in downstream analysis as their error exceeded half of the mean value. (c) Histograms of all fitted transit times. Data was fit to

a Gaussian model to identify a main and a tail population, characterizing heterogeneity in enzyme diffusion. A tail is most strongly observed in the initial 30 minutes of the reaction, likely representing slowed-down enzyme docked onto substrate. (d) Analysis of the diffusion coefficients for both main and tail populations reveal a slight increase in diffusion among free enzyme over time. For the segregated populations, the ratio of diffusion coefficients under STED to those under confocal inform of anomalous behavior, with ratio << 1 indicative of subdiffusive behavior, and ratio ~ 1 corresponding to free diffusion. Some recovery towards free diffusion is observed over the reaction timecourse, primarily in the main component.

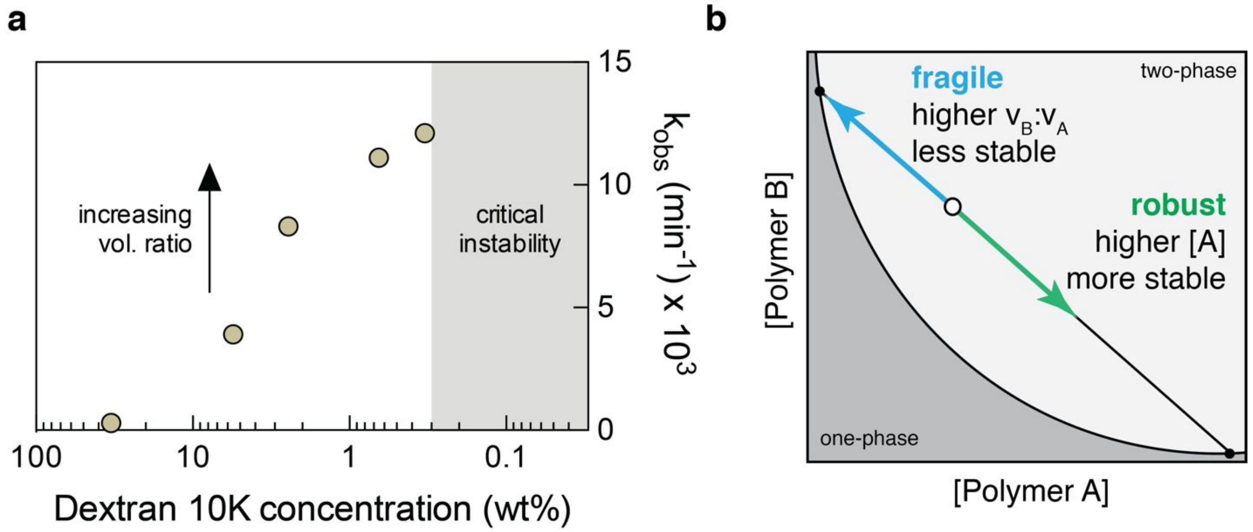

Supplementary Figure 12. Increasing concentration is a robust control alternative to the established fragile method of increasing volume ratio and partitioning. (a) Using published data[10], we plotted the first-order rate constants for a ribozyme compartmentalized within a PEG-dextran two-phase system supplemented with 10 mM Mg^{2+}, against overall dextran concentrations in the system. Increasing volume ratio, meaning smaller dextran phases, corresponded with higher observed rates k, but this would be considered a fragile control method: maximal enzyme performance would mean designing the phase-separating system to be near the phase boundary, which is sensitive to small fluctuations and can be unstable. (b) Schematic showing the difference in strategy for prior work and this study. Increasing volume ratios are found by moving along the tie line towards the phase boundary. Increasing concentrations of one or both components would mean movement well into the stable two-phase region. In our work, increasing concentrations in a coacervate system also corresponded with lower viscosity, suggesting that allowing for faster diffusion can complement or even supersede the benefits of having small compartment sizes.

Supplementary References

1 Waithe D., Schneider F., Chojnacki J., Clausen M. P., Shrestha D., de la Serna J. B. & Eggeling C. Optimized processing and analysis of conventional confocal microscopy generated scanning FCS data. *Methods* **140-141**, 62-73, doi:10.1016/j.ymeth.2017.09.010 (2018).

2 Honigmann A., Mueller V., Ta H., Schoenle A., Sezgin E., Hell S. W. & Eggeling C. Scanning STED-FCS reveals spatiotemporal heterogeneity of lipid interaction in the plasma membrane of living cells. *Nat. Commun.* **5**, doi:10.1038/ncomms6412 (2014).

3 Digman M. A., Sengupta P., Wiseman P. W., Brown C. M., Horwitz A. R. & Gratton E. Fluctuation Correlation Spectroscopy with a Laser-Scanning Microscope: Exploiting the Hidden Time Structure. *Biophys. J.* **88**, L33-L36, doi:10.1529/biophysj.105.061788 (2005).

4 Yang L., Zhou N. & Tian Y. Purification, characterization, and biocatalytic potential of a novel dextranase from Chaetomium globosum. *Biotechnol. Lett.* **40**, 1407-1418, doi:10.1007/s10529-018-2599-z (2018).

5 Lehninger A. L., Nelson D. L. & Cox M. M. *Lehninger principles of biochemistry*. 5th edn, (W.H. Freeman, 2008).

6 Huang R., Zhong L., Xie F., Wei L., Gan L., Wang X. & Liao A. Purification, Characterization and Degradation Performance of a Novel Dextranase from Penicillium cyclopium CICC-4022. *Int. J. Mol. Sci.* **20**, doi:10.3390/ijms20061360 (2019).

7 Richards G. N. & Streamer M. Studies on dextranases. *Carbohydr. Res.* **32**, 251-260, doi:10.1016/s0008-6215(00)82103-5 (1974).

8 Wang H., Lin Q., Dong D., Xu Y., Liu M., Lu J., Lyu M. & Wang S. Cloning of Cold-Adapted Dextranase and Preparation of High Degree Polymerization Isomaltooligosaccharide. *Catalysts* **12**, doi:10.3390/catal12070784 (2022).

9 Gennes P. G. d. *Scaling concepts in polymer physics*. (Cornell University Press, 1979).

10 Strulson C. A., Molden R. C., Keating C. D. & Bevilacqua P. C. RNA catalysis through compartmentalization. *Nat. Chem.* **4**, 941-946, doi:10.1038/nchem.1466 (2012).
